# Super determinant1A, a RAWULdomain-containing protein, modulates axillary meristem formation and compound leaf development in tomato

**DOI:** 10.1093/plcell/koab121

**Published:** 2021-05-01

**Authors:** Hernán López, Gregor Schmitz, Rahere Thoma, Klaus Theres

**Affiliations:** Max Planck Institute for Plant Breeding Research, Cologne D-50931, Germany

## Abstract

Shoot branching and complex leaf development relies on the establishment of boundaries that precedes the formation of axillary meristems (AMs) and leaflets. The tomato (*Solanum lycopersicum*) *super determinant* mutant is compromised in both processes, due to a mutation in *Sde1A*. *Sde1A* encodes a protein with a RAWUL domain, which is also present in Polycomb Group Repressive Complex 1 (PRC1) RING finger proteins and WD Repeat Domain 48 proteins. Genetic analysis revealed that *Sde1A* and *Bmi1A* cooperate, whereas *Bmi1C* antagonizes both activities, indicating the existence of functionally opposing PRC1 complexes that interact with Sde1A. *Sde1A* is expressed at early stages of boundary development in a small group of cells in the center of the leaf-axil boundary, but its activity is required for meristem formation at later stages. This suggests that Sde1A and Bmi1A promote AM formation and complex leaf development by safeguarding a pool of cells in the developing boundary zones. Genetic and protein interaction analyses showed that *Sde1A* and *Lateral suppressor* (*Ls*) are components of the same genetic pathway. In contrast to *ls*, *sde1a* mutants are not compromised in inflorescence branching, suggesting that *Sde1A* is a potential target for breeding tomato cultivars with reduced side-shoot formation during vegetative development.

## Teaser

The RAWUL protein-encoding gene *Super determinant1A* interacts genetically with the *Bmi1* family and plays a crucial role at early steps of axillary meristem formation and complex leaf development in tomato.

## Introduction

The remarkable variation in plant architecture is driven primarily by the degree of branching during vegetative and reproductive development. In seed plants, shoot branches develop from established axillary meristems (AMs), an evolutionary innovation that underpinned diversification in shoot architecture (Sussex and Kerk, 2001; Langdale and Harrison, 2008). During the vegetative growth phase, AMs initiate at leaf axils that are located far from the shoot apical meristem (SAM; Greb et al., 2003; Xin et al., 2017; Ponraj and Theres, 2020). This initiation requires the establishment of cells within the leaf-axil boundary domain that have the competence to form new meristems. AMs develop into axillary buds (ABs) and their subsequent development requires the integration of whole-plant signaling, leading to dormant ABs or actively growing side shoots (Domagalska and Leyser, 2011; Martín-Fontecha et al., 2018; Barbier et al., 2019).

The establishment of a boundary domain between the pluripotent stem cells of the SAM and the cells specified for leaf organogenesis precedes the formation of AMs (Wang et al., 2016). Boundary cells show characteristic morphological features, such as small size, infrequent divisions, a parallel arrangement of microtubules, and rigid cell walls (Hussey, 1971; Reddy et al., 2004; Burian et al., 2016). At the molecular level, boundary cells express evolutionarily conserved genes common to pathways that regulate SAM formation (Tian et al., 2014, 2019). For example, central players in boundary establishment and SAM initiation in numerous plant species are orthologs of the NAC-domain transcription factor genes *CUP-SHAPED COTYLEDON1* (*CUC1*), *CUC2*, and *CUC3* in Arabidopsis (*Arabidopsis thaliana*) and *Goblet* (*Gob*) in tomato (*Solanum lycopersicum*; Aida et al., 1997; Vroemen et al., 2003; Blein et al., 2008; Raman et al., 2008; Berger et al., 2009), which in turn are posttranscriptionally controlled by the conserved miRNA miR164 (Nikovics et al., 2006). The GRAS transcription factor LATERAL SUPPRESSOR (LAS in Arabidopsis and Ls in tomato) functions downstream of CUC/Gob (Schumacher et al., 1999; Greb et al., 2003; Raman et al., 2008; Rossmann et al., 2015). Furthermore, a parallel pathway containing the MYB-domain transcription factors REGULATOR OF AXILLARY MERISTEMS1 (RAX1), RAX2, and RAX3 in Arabidopsis and Blind (Bl) in tomato controls AM initiation (Schmitz et al., 2002; Keller et al., 2006; Müller et al., 2006; Busch et al., 2011).

In species with compound leaves, such as tomato, the regulatory mechanisms that operate during leaf primordium initiation and boundary domain establishment are also recruited for leaflet development. For instance, the leaflet boundary domain is specified by the AM regulators Ls, Gob, and the Bl paralog Potato leaf (Busch et al., 2011). In mature leaves of tomato and other species, these boundaries can form ectopic meristems (EMs) that have the potential to develop into shoots (Rossmann et al., 2015).

The establishment and maintenance of cell pools that are competent to form meristems require the specific activation and repression of transcriptional programs, which involve the spatiotemporal deployment of epigenetic mechanisms that operate on chromatin. One group of enzymes that alters DNA accessibility and exposes cis-regulatory elements to transcription factors is composed of chromatin-remodeling factors. In Arabidopsis, two such factors are the Sucrose Non-Fermentable (SNF2)-type chromatin-remodeling ATPases SPLAYED and BRAHMA. During embryogenesis, both proteins promote the transcription of *CUC* genes at the cotyledon boundary (Kwon et al., 2006), whereas in the postembryonic phase, SPLAYED promotes the expression of the SAM regulator *WUSCHEL* (*WUS*; Kwon et al., 2005). It is currently unknown whether such a mechanism also operates during AM formation. However, a transcriptionally permissive environment at the shoot meristem regulator *SHOOT MERISTEMLESS* (*STM*) locus is maintained in young leaf axils (Cao et al., 2020), whereas the *WUS* locus is characterized by a transcriptionally restrictive environment that gradually becomes transcriptionally permissive during early stages of AM initiation (Wang et al., 2017), indicating the existence of different phases of epigenetic regulation during successive stages of AM formation.

The transcriptional repression of regulatory genes that control the commitment of cells to differentiate is thought to play a major role within the boundary domain, to maintain cellular pluripotency (Wang et al., 2016). Polycomb Group (PcG) proteins are key transcriptional repressors and assemble into two major complexes known as PcG Repressive Complex 1 (PRC1) and PRC2: PRC1 regulates Histone H2A lysine 119 (H2AK119, H2AK121 in Arabidopsis and tomato) monoubiquitylation, while PRC2 regulates Histone H3 lysine 27 (H3K27) trimethylation. PRC1 and PRC2 recruitment and their mechanistic role during the repression of gene expression is an active area of research; however, there remains a profound lack of knowledge concerning their roles, as well as the relative contributions of their individual components during all stages of AM formation. Here, we show that *Super determinant 1A* (*Sde1A*) shares an evolutionary origin with the B cell-specific Moloney murine leukemia virus integration site 1 (Bmi1) constituents of PRC1, and that it operates at the early stages of boundary development. Sde1A is required to maintain high expression levels of the shoot meristem regulator genes *Tomato Knotted 2* (*TKn2*, the tomato *STM* ortholog) and *CLAVATA3* (*CLV3*), as well as the boundary regulators *Gob*, *Ls*, and *Bl*. Together with Bmi1A, Sde1A increases the morphogenetic competence of leaf axils and leaves and promotes the formation of AMs, leaflets, and EMs, indicating the presence of an active epigenetic regulation during the establishment of the leaf-axil boundary domain. In contrast, Bmi1C antagonizes Sde1A and Bmi1A activity, implying that in tomato opposing PRC1 complexes promote or inhibit meristematic competence in combination with Sde1A. Furthermore, protein and genetic interaction analyses indicate that this mechanism requires the activity of the AM regulator Ls.

## Results

### The *sde* mutant shows reduced shoot branching and reduced leaf dissection

**Figure 1:**
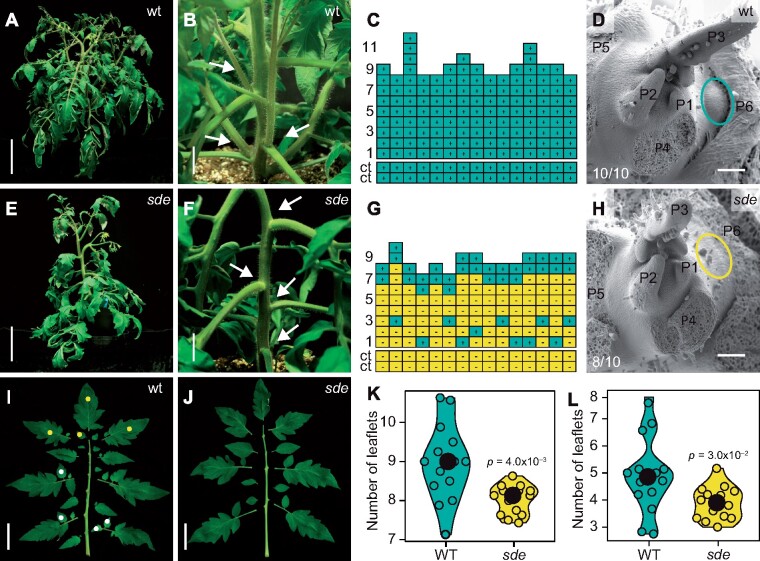
*sde* is compromised in shoot branching and leaf dissection. A and E, Growth habit of wt (A) and *sde* (E) plants 8 weeks after sowing. B and F, Close-up of leaf axils from plants shown in A (B) and E (F), respectively. Arrows highlight filled leaf axils in (B) and empty leaf axils in F. C and G, Graphic representation of AB formation. Each column represents a single plant and each square within a column denotes a single leaf axil. Green indicates the presence of an AB or a side shoot, yellow indicates an empty leaf axil. Cotyledons are indicated as ct. D and H, Scanning electron micrographs of vegetative shoot apices (14-d-old seedlings) including the P6 leaf axil. The ovals delimit P6 axils developing an AM in wt (D), and an empty leaf axil in *sde* (H). I and J, Picture of leaf no. 6 of wt (I) and *sde* (J) plants showing leaflets separated from the rachis. Examples of first-order (yellow) and second-order (white) leaflets are indicated in I. K and L, Violin plots of the mean number of first-order (K) and second-order (L) leaflets per leaf along the primary shoot (leaves 1–8) in wt and *sde* plants. Median values are indicated by a black dot. *n* = 14 in K and L. *P*-values were determined by two-tailed, two-sample *t* test. Bars represent 15 cm in (A) and (E), 4 cm in (B) and (F), 50 µm in (D) and (H), and 5 cm in (I) and (J).

The *sde* mutant of tomato is characterized by an altered development of AMs. Under greenhouse conditions, all leaf axils of wild-type (wt, VF36 cultivar) plants developed ABs, whereas ABs failed to develop in *sde* during early to mid-vegetative development (∼leaf axils 1–6; Figure 1, A–C and E–G). The leaf axils located close to the first inflorescence were not affected and produced ABs (Figure 1G). This trait was fully penetrant in greenhouse and controlled environment conditions, but its expressivity varied from 13% to 90% (Supplemental Figure S1). To discriminate between AM initiation and outgrowth, we scored shoot apices under a stereomicroscope and imaged them by scanning electron microscopy (SEM). During the vegetative phase (2-week-old seedlings), the axils of young leaf primordia (P) 1–5 were barren in wt and *sde*. In wt, axils of leaf primordia older than P6 developed bulges as the first morphological indication of AM initiation, but these were absent in *sde* (Figure 1, D and H). During reproductive development (4-week-old plants), wt shoot apices developed ABs at all leaf axils (Supplemental Figure S2, A and B), whereas *sde* had empty leaf axils (45%), a disorganized mass of cells (11%), ABs that were delayed in development (25%), or fully developed ABs (19%, Supplemental Figure S2, C–F).

Tomato plants alternate between vegetative and reproductive growth, which is known as sympodial growth. The primary SAM, which originates during embryogenesis, produces between 8 and 12 leaves before transitioning to reproductive growth as an inflorescence meristem (IM). Vegetative growth subsequently proceeds via sympodial shoots that develop from the uppermost AM. Each sympodial shoot produces three leaves before transitioning to reproductive growth in a continuously reiterated process in indeterminate tomato cultivars. Determinate tomato cultivars harbor a mutation in the *Self pruning* (*Sp*) gene and show a progressive reduction in the number of leaves formed by sympodial meristems (Pnueli et al., 1998), which is a desirable trait for tomato field varieties because it facilitates mechanical harvesting. The VF36 cultivar harbors the *sp* allele and expresses this distinctive *sp* phenotype. The *sde* mutation enhanced the *sp* phenotype, which culminated in sympodial shoots that bypassed vegetative growth and developed as inflorescences, to generate plants with two consecutive inflorescences (Supplemental Figure S3, A–C). This trait showed genotype-by-environment interaction and incomplete penetrance (Supplemental Figure S3, C and D), but the flowering time of the primary shoot and the number of flowers per inflorescence in *sde* mutants were not affected (Supplemental Figure S3, E and F).

To monitor differences in compound leaf development between *sde* and wt, we assessed the mean number of first- and second-order leaflets for all leaves of the primary shoot (leaves1–8) in 12-week-old plants. About nine first-order leaflets per leaf developed in wt plants, whereas *sde* possessed eight (Figure 1, I–K). Similarly, wt plants developed five second-order leaflets on average, whereas *sde* developed four (Figure 1, I–L), which represented a slight but significant reduction in leaflet number in *sde*.

### Molecular cloning of *Sde1A*

**Figure 2:**
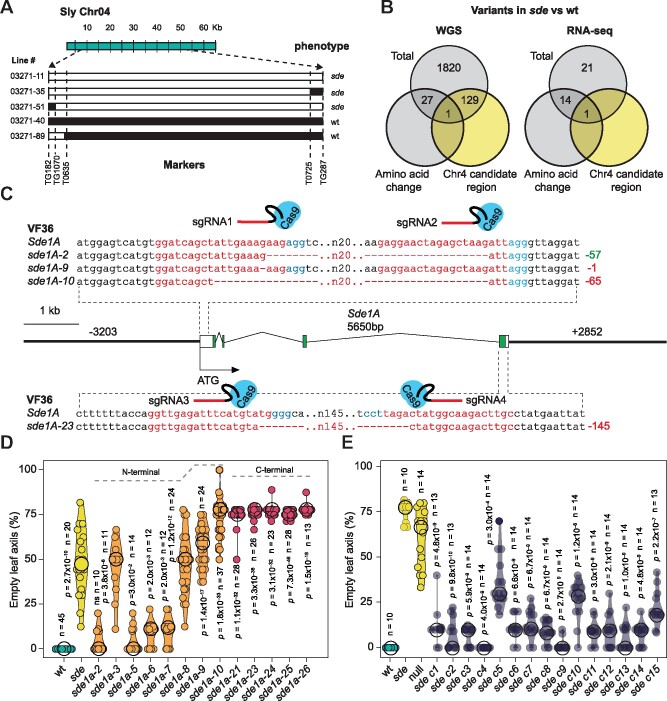
*Sde1A* encodes a RAWUL-domain protein. A, *Sde1A* maps to a region on chromosome 4 delimited by markers T0635 and T0725. Chromosome 4 from the tomato reference strain Heinz is represented at the top (green). Individual recombinant chromosomes are shown below for the recombinant lines indicated on the left and their corresponding phenotype on the right. White segments denote genomic segments inherited from *sde*; black segments represent genomic segments inherited from *S. pennellii*. B, Venn diagrams showing the total number of DNA variants, variants that cause an amino acid change and variants located within the chromosome 4 candidate region, in a comparison between VF36 and *sde* by whole-genome and RNA-sequencing (WGS and RNA-seq), respectively. C, Schematic representation of the *Sde1A* locus indicating the genomic region used for complementation and sites targeted by CRISPR-Cas9. The promoter and the terminator are indicated as thick black bars. The conserved (RAWUL) and the nonconserved coding region are indicated in green and white, respectively. sgRNA sequences are represented in red, followed by the adjacent protospacer motif in blue. Partial DNA sequences of individual deletions in the first and fourth exons are shown. The distance between the shown adjacent sequences is denoted by *n*. The size of the in-frame (green) and frameshift (red) deletions is indicated on the right in bp. D, Violin plots of shoot branching phenotype (% empty leaf axils) of *Sde1A* CRISPR-Cas9 mutants carrying independent mutations in the VF36 background. E, Violin plots of shoot branching phenotype (% empty leaf axils) of different T_1_ plants harboring an *Sde1A* genomic fragment that complements the *sde* mutant phenotype. Median values are indicated by a black circle. *n* values in (D) and (E) represents the number of individual plants. *P*-values were determined by two-tailed, two-sample *t* tests against wt in (D) and against the sister plants lacking the transgene (null) in (E).

A backcross F_2_ population (BCF2) revealed that the *sde* phenotype segregated as a monogenic recessive trait (Supplemental Figure S4A) and the underlying mutation, referred to as *sde1a*, was characterized in more detail. Using an F_2_ population from a cross between *sde* and the wild tomato species *Solanum pennellii*, we mapped *Sde1A* to a region between markers T635 and T725 on chromosome 4 (Figure 2A). Due to low recombination frequency over this interval, the *sde1a* mutation could not be identified by conventional genetic mapping. Therefore, we followed comparative whole-genome sequencing (WGS) and transcriptome deep sequencing (RNA-seq) approaches. Data from WGS identified 1,977 variants that were evenly distributed throughout the genome of the *sde* mutant, of which 130 were within the *sde1a* candidate region (Figure 2B, Supplemental Figure S5A, Supplemental Data Set S1). RNA-seq analysis of vegetative shoot apices revealed no differences in the expression of genes within the target region (Supplemental Data Set S2), indicating that the *sde1a* mutation does not influence transcript levels, but might impact protein activity. The combination of genome resequencing and RNA-seq analysis uncovered a unique candidate mutation that also fully cosegregated with the *sde* phenotype in the BCF2 population (Supplemental Figure S4, A and B, Supplemental Data Set S3). A transversion of a guanine to a thymidine nucleotide (251G>T) in the gene Solyc04g049190, referred to as *Sde1A* hereafter, led to an amino acid exchange of a tryptophan to a leucine residue (W84L) in the encoded protein (Figure 2B, Supplemental Figure S5B, Supplemental Data Set S4). Analysis of the mapped RNA-seq reads in combination with cDNA cloning guided the reannotation of *Sde1A*, which encodes a ring-finger and WD40-associated ubiquitin-like protein (RAWUL, Supplemental Figure S6). The RAWUL domain is present in the C-terminal region of RING finger proteins from the PRC1 complex (Ring1 and Bmi1) and in the C-terminal region of WD-repeat 48 (Wdr48) proteins (Sanchez-Pulido et al., 2008). The tomato genome contains five genes that encode RING-finger and RAWUL-domain proteins, two of which correspond to *Ring1* genes (*Ring1A*: Solyc02g077890 and *Ring1B*: Solyc07g053800) and three to *Bmi1* genes (*Bmi1A*: Solyc09g065990, *Bmi1B*: Solyc06g008600, and *Bmi1C*: Solyc06g084040). Furthermore, we identified two Wdr48-encoding genes (*Wdr48A*: Solyc01g098090 and *Wdr48B*: Solyc08g023570) and an additional *Sde1A* paralog (*Sde1B*: Solyc01g009720) in tomato, all of which contain a C-terminal RAWUL domain. However, none of these have been functionally characterized. In the PRC1 complex, Ring1 and Bmi1 interact via the N-terminal RING-finger domain, whereas the RAWUL domain interacts with additional PRC1 core proteins and leads to the formation of functionally distinct PRC1 complexes (Kim, 2017). Recently, the *Sde1A* homologous genes *LAX PANICLE2* (*LAX2*, Tabuchi et al., 2011) and *barren stalk2* (*ba2*, Yao et al., 2019) from rice (*Oryza sativa*) and maize (*Zea mays*), respectively, were shown to regulate shoot branching. However, Basic Local Alignment Search Tool for Protein (BLASTP) searches (Altschul et al., 1997) and protein alignment analysis identified Os12g0479100 and GRMZM2G447297 as the closest Sde1A homologs (Supplemental Figure S7) from rice and maize, respectively, whereas Sde1B is the putative ortholog of LAX2 and ba2. An analysis of Sde1A through Protein Variation Effect Analyzer (PROVEAN, Choi and Chan, 2015) indicated that amino acid W84 located within the RAWUL domain is highly conserved among Sde1 and Bmi1 homologs, suggesting that the W84L amino acid exchange might have a deleterious effect on protein activity.

To obtain additional independent *sde1a* mutant alleles, we edited the *Sde1A* locus using genome editing via Clustered Regularly Interspaced Short Palindromic Repeats (CRISPR) and CRISPR-associated nuclease (Cas9; Belhaj et al., 2013). We designed two pairs of single guide RNAs (sgRNAs) targeting either the first or the fourth exon of *Sde1A* to alter either the encoded nonconserved region or the conserved RAWUL domain, respectively (Figure 2C;Supplemental Figure S8). Four out of eight independent mutations in the first exon and all five mutations in the last exon recapitulated the *sde* branching phenotype (Figure 2D;Supplemental Figure S8). In addition, transformation of *sde* with a *Sde1A* genomic fragment functionally complemented the *sde* mutant (Figure 2E). Taken together, three independent approaches validated the *sde1a* mutation as being causal for the *sde* phenotype.

To assess the role of *Sde1A* in the regulation of AM formation in different genetic backgrounds, we crossed *sde* to the greenhouse cultivar Moneymaker and the field variety M82. Furthermore, we generated additional *Sde1A* genome-edited alleles in Moneymaker. Compared to the VF36 BCF2 population, F_2_ populations from crosses between *sde* and Moneymaker or M82 showed a lower proportion of plants displaying branching defects (∼13% and ∼3%, respectively) that indicated a strong but variable degree of epistasis (Supplemental Figure S4A). Sanger sequencing of the *Sde1A* locus showed that F_2_ plants with AM initiation defects comparable to *sde* are homozygous for *sde1a* (Supplemental Figure S4A), indicating that the *sde* phenotype is dependent on *sde1a*. Moreover, the *sde1a* allele co-segregated with defects in AM formation in 98% and 93% of all F_2_ plants that showed AM initiation defects from the crosses between *sde* and Moneymaker or M82, respectively (Supplemental Figure S4B). In contrast, new *sde1a* alleles generated in Moneymaker showed a small difference in the pattern of shoot branching compared to that of wt plants (e.g. *sde1a-16*, Supplemental Figure S8, A and C). However, genetic analysis of *sde1a-16* mutant plants in a mixed MM/VF36 genetic background showed a stronger defect on AM formation than the original *sde* mutant (Supplemental Figure S8, B and C). This result indicated that *sde1a-16* is indeed a loss-of-function allele and that *sde1a* mutants require VF36 modifiers to display the *sde* phenotype.

### *Sde1A* is specifically expressed at the center of the leaf-axil boundary, whereas *Sde1B* is broadly expressed

**Figure 3:**
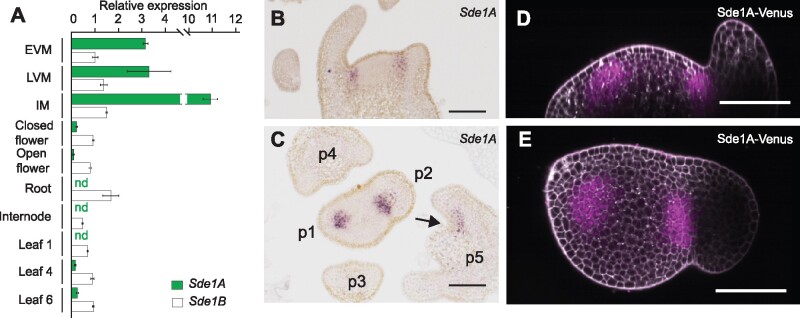
*Sde1A* is preferentially expressed in shoot meristematic tissues. A, RT-qPCR analysis of *Sde1A* and *Sde1B* relative transcript levels in early vegetative meristems (EVM), late vegetative meristems (LVM), inflorescence meristems (IM), closed flowers, open flowers, roots, internodes, and leaves 1, 4, and 6. The expression level of *GAPDH* was used as a reference and was set to one. *Sde1A* transcripts were not detected (nd) in roots, internodes or in the oldest leaf no. 1, which is the leaf that is furthest away from the SAM. B, C, RNA in situ hybridization analysis of longitudinal sections (B) and cross-sections (C) through vegetative shoot apices hybridized with an *Sde1A* antisense probe. D and E, Confocal images of a line harboring a *Sde1A-Venus* translational reporter construct. D shows an optical transverse section and E a longitudinal section through a representative shoot apex. Bars in (A) represent the standard error of the mean from three technical replicates. The experiment was repeated twice with similar results. Bars in (B–E) represent 100 µm.

The mRNA of *Sde1A* predominantly accumulated in the SAM at the vegetative, transition and inflorescence stages, whereas that of *Sde1B*, the closest *Sde1A* homolog, was present at low levels in all tissues analyzed (Figure 3A). Furthermore, the analysis of tissue-specific *Sde1A* expression by RNA in situ hybridization revealed a defined expression domain in the center of the boundary region between the emerging leaf primordium and the SAM, as well as in the presumptive leaflet boundary domain in older leaf primordia (P5, Figure 3, B and C). We observed this expression domain in P1 and P2, whereas *Sde1A* signal was weak or absent in older leaf axils (Figure 3, B and C; Supplemental Figure S9A). Longitudinal sections revealed that the *Sde1A* expression domain extends approximately six to seven cell layers in depth, starting from cell layer 2 (Figure 3B). Transverse sections showed that the *Sde1A* expression domain encompasses about five cell layers along the proximodistal axis (meristem to leaf) and eight cell layers along the lateral axis (Supplemental Figure S9A). This pattern extended through five consecutive sections, creating an ellipsoid-shaped expression domain. We did not detect *Sde1A* transcripts during the early stages of AM formation, first distinguished as bulges in P6–P7 axils, indicating that *Sde1A* expression is not required at those stages (Supplemental Figure S9A). To compare the patterns of *Sde1A* transcript and Sde1A protein localization, we generated a translational fusion between *Sde1A* and *Venus* driven by the *Sde1A* promoter (*Sde1A-Venus*). Three independent transgenic lines showed that Sde1A-Venus accumulates in a domain highly similar to that of the *Sde1A* transcript, indicating that the Sde1A protein does not move (Figure 3, D and E). In contrast to *Sde1A*, RNA in situ hybridization for *Sde1B* revealed a broad expression in the SAM and leaf primordia (Supplemental Figure S9B).

### Ectopic expression of *Sde1A* increases morphogenetic competence in leaves

**Figure 4:**
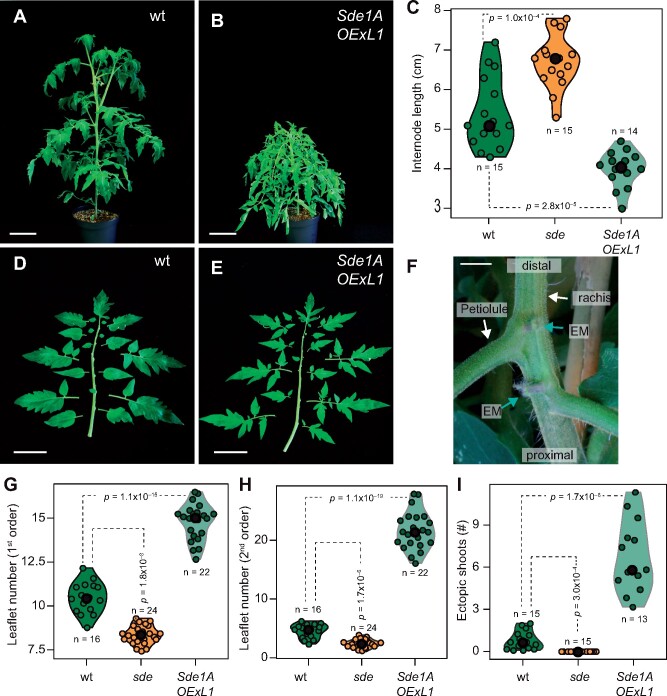
Ectopic expression of *Sde1A* increases morphogenetic competence in tomato leaves. A and B, Growth habit of a wt (A) and a plant overexpressing *Sde1A* (*Sde1A-OExL1*, B). C, Violin plot of internode length of wt, *sde*, and *Sde1A-OExL1* plants. D, E, Comparison of leaf complexity in representative leaves (leaf no. 6) of wt (D) and *Sde1A-OExL1* (E) plants. F, Ectopic shoot formation in a leaf of an *Sde1A-OExL1* plant. G–I, Violin plot of the number of first-order (G) and second-order (H) leaflets and the number of ectopic shoots on leaves (I) of wt, *sde*, and *Sde1A-OExL1* plants. Median values are indicated by a black circle. *n* values in (C) and (G–I) represent the number of individual plants. *P*-values were determined by two-tailed, two-sample *t* tests.

To test whether *Sde1A* can promote meristem formation, an activity associated with the boundary domain, we expressed *Sde1A* under the Arabidopsis *UBIQUITIN10* (*UBI10*) promoter in wt VF36. Out of 15 independent transgenic tomato lines, we selected three with increased *Sde1A* transcript levels (Supplemental Figure S10, A and B). All three lines showed similar developmental phenotypes (Supplemental Figure S10, C and D). A decrease in plant size due to reduced internode elongation in *Sde1A-OExL1* plants correlated with increased *Sde1A* expression, which is consistent with the longer internodes seen in the *sde* mutant (Figure 4, A–C). The line *Sde1A-OExL1*, which showed the highest *Sde1A* mRNA levels, was selected for detailed characterization.

To assess differences in leaf morphogenetic competence, we monitored changes in leaf complexity (leaflet number per leaf) and EM formation in *Sde1A-OExL1* plants. On average, wt plants developed between 11 and 5 first- and second-order leaflets per leaf, respectively (Figure 4, D, G, and H). In comparison, the number of first-order leaflets in *Sde1A-OExL1* was 41% higher, while it was 20% lower in *sde*. Similarly, *Sde1A-OExL1* plants had almost four-fold more second-order leaflets than wt, whereas *sde1* plants had 46% fewer (Figure 4, D, E, G, and H). Typically, tomato leaves form EMs at the distal leaflet boundary (Rossmann et al., 2015) with a mean of approximately one EM per leaf in wt VF36 plants (Figure 4I). In *Sde1A-OExL1* plants, about six EMs were present per leaf, whereas we observed none on *sde* leaves (Figure 4, F and I). Moreover, EMs in some *Sde1A-OExL1* plants were not limited to the petiolule–rachis junction, and when the plants had grown to maturity, EMs developed into shoots that covered the entire leaf (Figure 4F;Supplemental Figure S10, E and F).

### *Sde1* originated in the last common ancestor of embryophytes and shares part of the *Bmi1* exon–intron structure, suggesting a common evolutionary history

**Figure 5:**
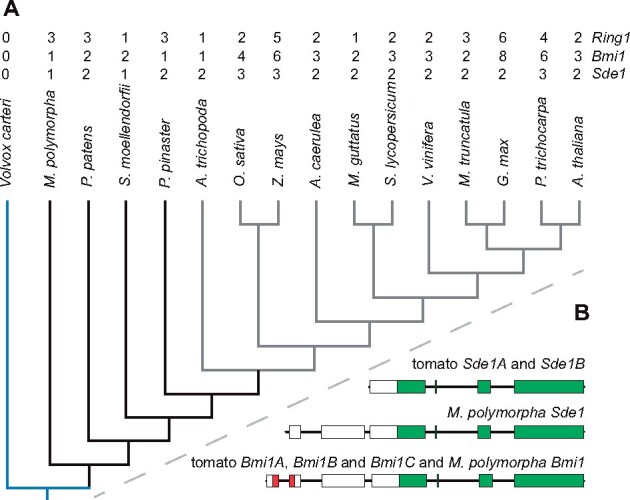
Exon–intron structures of *Sde1* and *Bmi1* genes suggest they share a common evolutionary history. A, Phylogenetic relationships among embryophytes and representative members of chlorophytes, liverworts, mosses, lycophytes, gymnosperms, and angiosperms. Chlorophytes are represented in blue, basal plants in black and flowering plants in gray. The copy number of *Sde1*, *Bmi1*, and *Ring1* genes are indicated above. The tree was redrawn based on the relationship of the respective sequenced genomes deposited in Phytozome. B, Gene models of *Sde1*- and *Bmi1*-related genes from tomato and *Marchantia polymorpha*. Red boxes denote the region encoding the C3HC4 ring-finger domain; green boxes represent the region encoding the RAWUL domain.

BLASTP searches identified 138 Sde1A homologs, which were all restricted to embryophytes. The liverwort *Marchantia polymorpha* and the lycophyte *Selaginella moellendorffii* each contained a single gene with similarity to *Sde1*, whereas the genome of the moss *Physcomitrium* (*Physcomitrella*) *patens* contained two genes with similarity to *Sde1* (Figure 5A). The genome from the extant relative of flowering plants *Amborella trichopoda*, the gymnosperm *Pinus pinaster*, and that of most of the sampled dicots (e.g. Arabidopsis and tomato), contained two *Sde1*-like genes (Figure 5A;Supplemental Data Set S5). Grasses (e.g. rice and maize), however, usually contained three *Sde1*-like genes (Figure 5A;Supplemental Data Set S5). Using a similar approach for proteins containing a RAWUL domain, we identified 260 Bmi1, 143 Ring1, and 102 Wdr48 homologous proteins in embryophytes and animals, but none in chlorophytes or rhodophytes (Supplemental Data Set S5). A phylogenetic reconstruction (Jones et al., 1992) showed that Ring1-like and Wdr48-like proteins from animals and plants form closely related clades (Supplemental Figure S11A). In contrast, Bmi1-like proteins from animals and plants clustered within separate clades (Supplemental Figure S11A). Notably, Sde1-like proteins clustered with plant Bmi1-like proteins, suggesting that they share a common evolutionary origin.

The gene models of *Sde1* and *Bmi1* homologous genes from plants revealed structural conservation over the region encoding the RAWUL domain (Figure 5B). For example, the exon–intron borders and the conserved amino acid stretches were located at similar exonic positions in *Sde1-like* and *Bmi1*-like genes (Figure 5B;Supplemental Figure S11B). This conservation in gene structure, together with the protein similarity, suggested functional conservation between the RAWUL domains of Sde1-like and Bmi1-like proteins. To explore this possibility, we tested the interaction between Sde1A and known BMI1-interacting proteins by yeast-two hybrid (Supplemental Figure S12). This analysis showed that Sde1A interacts with Like heterochromatin protein 1 (Lhp1, Solyc01g081500). Notably, the mutant sde1a protein showed reduced interaction with Lhp1 (Supplemental Figure S12). In *Nicotiana benthamiana* mesophyll cells Lhp1 interacted with Sde1A (Supplemental Figure S12). The Lhp1 protein is a component of various PRC1 complexes (Gao et al., 2012; Forderer et al., 2016), indicating that Sde1A may be involved in the regulation of PRC1 activity by competing with Bmi1 for interacting partners or through the formation of noncanonical PRC1 complexes.

### *Sde1A* and *Bmi1A* synergistically promote AM formation, compound leaf development and EM formation, whereas *Bmi1C* antagonizes it

In the VF36 background, *sde1a* behaved as a recessive mutation; however, several genetic analyses suggested that VF36 is deficient in complementary pathways that regulate AM formation (sensitized background, Figure 7, E and F, Supplemental Figures S4, S8, and S19). Primary candidates that may compensate for the loss of *Sde1A* are *Sde1B* and the *Bmi1* genes (*Bmi1A*, *Bmi1B*, *Bmi1C*), which are also expressed in shoot apices (Supplemental Figure S13A). In Arabidopsis, complete loss of *BMI1* function leads to a significant reduction in the PRC1-associated mark H2A monoubiquitylation (H2AK121Ub), resulting in impaired plant development due to the upregulation of embryonic regulators such as *BABY BOOM* (*BBM*), *FUSCA3* (*FUS3*), and *LEAFY COLYLEDON1* (*LEC1*) and the meristem regulators *STM*, *WUS*, and *WUSCHEL-RELATED HOMEOBOX 5* (Xu and Shen, 2008; Bratzel et al., 2010).

To unravel the impact of Sde1 and Bmi1 on target loci, we compared the expression of known *Bmi1* targets and *sde1a* misexpressed genes in the tomato *sde1* and *bmi1* mutants. These experiments showed that individual *Sde1* and *Bmi1* genes collaborate in synergistic or antagonistic mode depending on the target locus. For instance, *TKn2* was upregulated in *bmi1b* and *bmi1c* mutants, whereas it was downregulated in *sde* and *sde1b*. *Wus* showed downregulation in *sde1b*, *bmi1a* and *bmi1b* mutants, whereas *Clv3* was downregulated in *sde* and *bmi1c* (Supplemental Figure S13B). Similarly, the AM regulators *Ls*, *Bl*, and *Gob* showed a coordinated mode of expression. For instance, *Ls* was upregulated in *bmi1a* and *bmi1b* mutants but downregulated in *sde*. *Gob* was upregulated in *bmi1b* and *bmi1c* mutants, whereas it was downregulated in *sde*, *sde1b*, and *bmi1a* and *Bl* was downregulated in all *sde1* and *bmi1* mutants (Supplemental Figure S13B). The *sde* RNA-seq analysis revealed misexpression of a gene encoding a proline-rich protein (Solyc10g007800) with similarity to PROTODERMAL FACTOR 1 (PDF1) from Arabidopsis, of the gene Solyc08g059700 and the genes Solyc04g081700 and Solyc09g064750 (Supplemental Data Set S2). Solyc08g059700 encodes a protein with similarity to KINASE-INDUCIBLE DOMAIN INTERACTING8 (KIX8) and KIX9 from Arabidopsis and the genes Solyc04g081700 and Solyc09g064750 encode two highly similar long noncoding RNAs. The gene homologous to *PDF1* was upregulated only in *sde*, whereas the lncRNAs were upregulated in *sde*, *sde1b*, and *bmi1a* mutants, and the *KIX8/KIX9* homolog was upregulated in all *sde1* and *bmi1* mutants (Supplemental Figure S13B).

**Figure 6:**
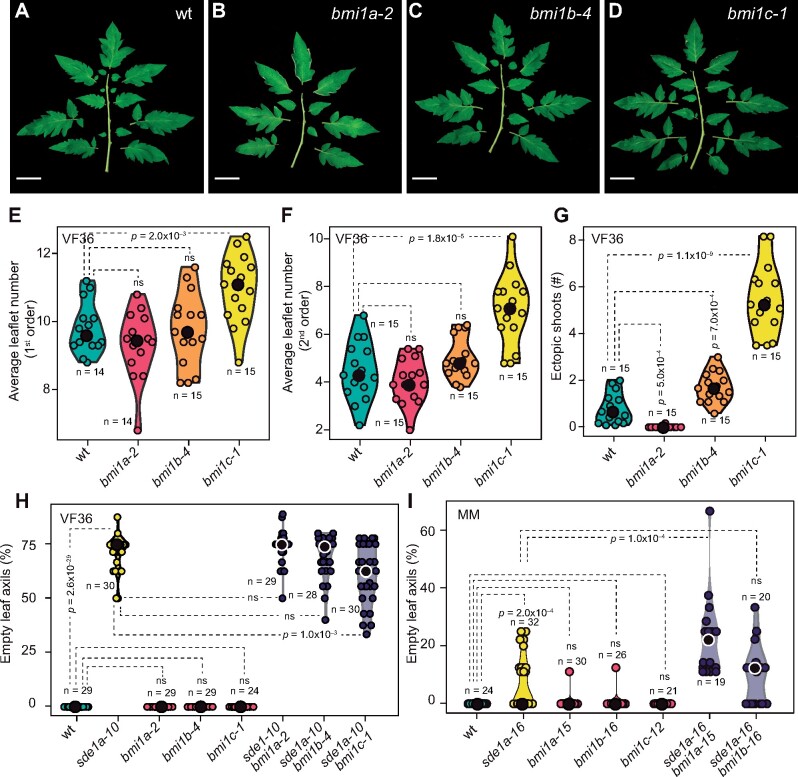
*Sde1A* and *Bmi1* genes show synergistic and antagonistic interactions. A–D, Comparison of phenotypes of representative leaf no. 6 of wt (A), *bmi1a-2* (B), *bmi1b-4* (C), and *bmi1c-1* (D) plants. E, F, Violin plots of the number of first-order (E) and second-order (F) leaflets of wt, *bmi1a-2*, *bmi1b-4*, and *bmi1c-1* plants. G, Violin plots of the mean number of ectopic shoots in wt, *bmi1a-2*, *bmi1b-2*, and *bmi1c-1* plants (G). H and I, Violin plots of the number of empty leaf axils (%) in *sde1a* in combination with *bmi1a*, *bmi1b*, or *bmi1c* mutant plants in a sensitized (VF36, H) and a nonsensitized (Moneymaker, I) *sde1a* background. Median values are indicated by a black or white circle. *n* values in (E–I) represent the number of individual plants. *P*-values were determined by two-tailed, two-sample *t* tests.

To test for genetic compensation or antagonism between *Sde1A*, *Sde1B*, *Bmi1A*, *Bmi1B*, and *Bmi1C* in the processes of meristem formation and complex leaf development, all genes were individually genome-edited using CRISPR-Cas9 technology in the VF36 and Moneymaker backgrounds (Supplemental Figures S14A, S15, A–C). This approach aimed to circumvent potential pleiotropic effects associated with a complete loss of PRC1 activity and consequently, an associated loss of transcriptional regulation at all stages of plant development. First, we analyzed the loss of function of *Sde1B*, the closest *Sde1A* homolog, in single and double mutant combinations. Similar to wt plants, *sde1b* mutants formed AMs. In addition, double mutant combinations showed no increase in the proportion of empty leaf axils during vegetative development compared to that in *sde1a* mutants, which suggests that these two genes do not function redundantly in AM formation (Supplemental Figure S14B). On the contrary, loss of function of individual *Bmi1* genes led to various developmental phenotypes, such as an increase in leaf complexity in *bmi1c* mutants and an increase in the number of EMs in *bmi1b* and *bmi1c* single mutants (Figure 6, A, D, E–G). In contrast, *bmi1a* mutants showed suppression of EM formation and a modest reduction in leaf complexity (Figure 6, A, B, E–G; Supplemental Figure S16, A–C). To test for genetic interactions between *Sde1A* and each *Bmi1* paralog, we generated double mutants by combining mutant alleles in the sensitized VF36 and the nonsensitized Moneymaker backgrounds. In VF36, loss of *Bmi1C* function partially suppressed the AM formation defects of one of the strongest CRISPR-Cas9 *Sde1A* loss-of-function alleles (*sde1a-10*; Figure 6H), whereas in Moneymaker, loss of *Bmi1A* function enhanced the weak *sde1a-16* defects in AM formation (Figure 6I). However, the increased leaf complexity and EM formation shown in the VF36 *bmi1c* mutant was suppressed in *sde1a bmi1c* (Supplemental Figure S16, A–C). Furthermore, although leaf complexity was not strongly reduced in *bmi1a* mutants, the *bmi1a bmi1c* double mutant also suppressed the increased leaf complexity and EM formation of *bmi1c*, indicating that both *Sde1A* and *Bmi1A* antagonize *Bmi1C* (Supplemental Figure S16, A–C). In accordance with this hypothesis, analysis of the *sde1a bmi1a* double mutant did not show a further decrease in leaf complexity compared to *sde1a*, indicating that *Sde1A* and *Bmi1A* may act in the same pathway to promote leaf complexity and meristem formation (Supplemental Figure S16, A–C). Taken together, this analysis indicates that *Sde1A* promotes various morphogenetic processes, whereas different *Bmi1* paralogs can cooperate to promote or inhibit them.

### Sde1A physically interacts with lateral suppressor

**Figure 7:**
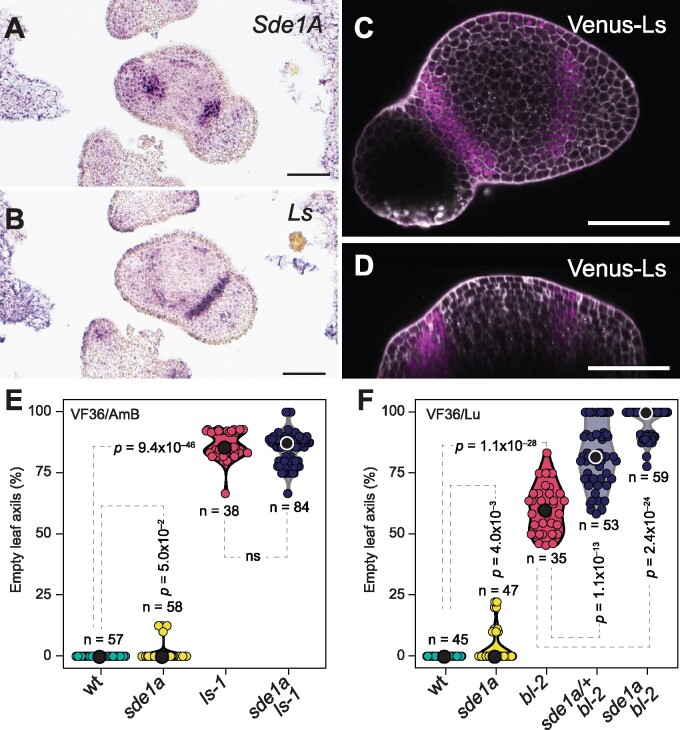
Sde1A and Lateral suppressor are components of a common genetic pathway that regulates AM initiation. A, B, RNA in situ hybridization of consecutive cross-sections of a vegetative shoot apex with an antisense *Sde1A* (A) or *Ls* (B) antisense probe. C and D, Confocal images of a line harboring an *Ls-Venus* translational reporter construct. C shows an optical transverse section and (D) a longitudinal section through a shoot apex. E, Violin plots of the percentage of empty leaf axils in wt, *sde1a*, *ls-1*, and *sde1a ls-1* plants in a VF36/Antimold B mixed background (VF36/AMB). F, Violin plots to compare the percentage of empty leaf axils in wt, *sde1a*, *bl-2*, *sde1a*/+ *bl-2*, and *sde1a bl-2* plants in a VF36/Lukullus mixed background (VF36/Lu). Median values are indicated by a black or white circle. *n* values in (E) and (F) represent the number of individual plants. *P*-values were determined by two-tailed, two-sample *t* tests. Bars in (A–D) represent 100 µm.

*Lateral suppressor* (*Ls*) and *Blind* (*Bl*) show overlapping expression domains with *Sde1A* in the leaf-axil boundary (Busch et al., 2011). Consecutive cross-sections revealed more precisely that this overlap is in the center of the boundary domain (Figure 7, A and B), suggesting that Ls and Bl might physically interact with Sde1A. To compare their accumulation patterns at the protein level, we generated a translational reporter whereby a *Venus-Ls* fusion is driven by the Ls promoter. Venus-Ls protein and *Ls* transcript accumulation strongly correlated with each other (Figure 7, B–D). In addition, *Sde1A* and *Ls* expression domains overlapped at the mRNA and protein levels (compare Figure 3, B–E with Figure 7, B–D). In yeast, Sde1A interacted with Ls and Bl but not with TKn2 (Supplemental Figure S17); however, in *N. benthamiana* mesophyll cells, Sde1A interacted only with Ls (Supplemental Figure S17). Ls interaction with the mutant sde1a protein was reduced, as shown by the limited growth of yeast cells in selective medium. Furthermore, deletion of a segment, including the N-terminal 17 amino acids of the RAWUL domain, or deletion of 39 amino acids of the RAWUL C-terminal domain, abolished Sde1A interaction with Ls, indicating that the RAWUL domain is required for the interaction with Ls (Supplemental Figure S17). In addition, Ls was able to interact with the RAWUL domain proteins Sde1B and Bmi1B, suggesting that different RAWUL domain proteins share similar interaction properties.

**Figure:**
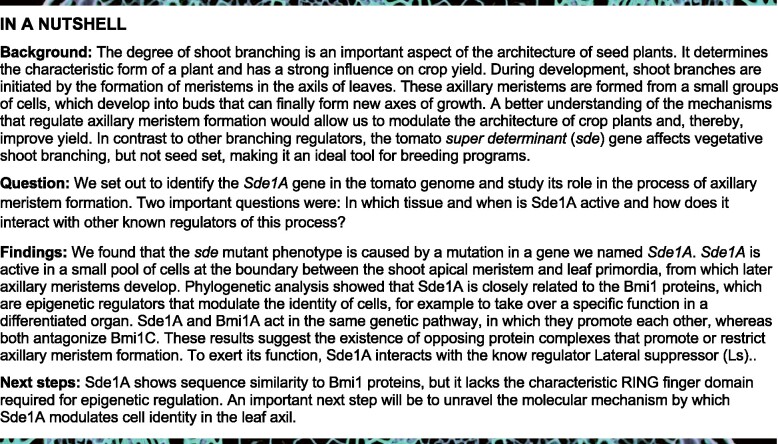


Genetic analysis showed that *sde1a ls-1* double mutants do no enhance the *ls-1* mutant phenotype (Figure 7E). In contrast, the *bl-2* AM formation defects were strongly enhanced in *sde1a bl-2* mutants in vegetative and reproductive stages and in a *sde1a* dose-dependent manner (Figure 7F). In 61% of *sde1a bl-2* double-mutant plants, AMs failed to form at any leaf axil of the primary shoot, including the uppermost leaf axils, which usually develop the sympodial shoot (Supplemental Figure S18); therefore, vegetative growth ceased and the life cycle of those plants terminated in a solitary flower.

Recently, the LAX2 and BA2 proteins in rice and maize were shown to interact with the basic Helix Loop Helix (bHLH) transcription factors LAX1 and BA1, respectively. In tomato, the orthologous bHLH gene *Uniflora* (*Uf*: Solyc09g005070) is a major regulator of inflorescence branching (Busch, 2011; Pier, 2015). However, during vegetative development, *Uf* mRNA was barely detectable (Supplemental Data Set S2) and *uf-1* mutants show no branching defects during the vegetative phase. Furthermore, *Uf* mutations did not enhance the *sde1a* defects in AM formation (Supplemental Figure S19). Taken together, these results indicate that *Sde1A* and *Ls* participate in a common genetic pathway that is independent of *Uf*, and that may function in parallel to the *Bl* pathway.

## Discussion

### *Sde1A* modulates AM initiation in tomato

Our findings demonstrate that *Sde1A* and *Bmi1* together shape plant development and provide a layer of regulation that operates at very early stages of AM formation and leaf development. During tomato vegetative development, AM formation manifests morphologically as bulges at P6–P7 leaf axils (Figure 1D), which correlates with the activation of the cell division marker Histone H4 in early P6 leaf axils (Rossmann, 2013) and indicates that AMs probably initiate in P5–P6 leaf axils. The *sde* mutant, which harbors a hypomorphic *sde1a* allele, is strongly compromised in AM formation (Figure 1; Supplemental Figures S2, S4, A and B). *Sde1A* accumulates in the center of the boundary domain in P1–P2 leaf axils, but not in the developing AMs (Figure 3; Supplemental Figure S9A), which indicate that *Sde1A* activity is required at early stages of boundary development to enable AM initiation at later developmental stages. Notably, *sde* can form a small number of functional AMs, implying that initiated AMs that develop beyond a critical point can continue normal development (Figure 1; Supplemental Figure S1). In Arabidopsis, the *RAX1* (Müller et al., 2006) and the *REGULATOR OF AXILLARY MERISTEM FORMATION* (Yang et al., 2012) genes show transient mRNA accumulation that ceases before AMs are initiated, similar to *Sde1A* expression in tomato. By contrast, the expression of the key AM formation regulators *Ls/LAS*, and *Gob/CUC* starts during early boundary development in tomato (Busch et al., 2011; Rossmann et al., 2015) and Arabidopsis (Greb et al., 2003; Raman et al., 2008) and is maintained until AMs initiate. The tomato *sde* mutant shows reduced mRNA levels of these boundary genes as well as of the meristem regulators *TKn2* and *CLV3*, suggesting that *Sde1A* activates AM formation in part by promoting high expression levels of these genes. In Arabidopsis leaf axils, a transcriptionally permissive epigenetic environment safeguards basal *STM* expression to allow for *STM* upregulation during AM initiation (Greb et al., 2003; Shi et al., 2016; Cao et al., 2020). The involvement of *Bmi1* genes, either cooperating or antagonizing *Sde1A* activity, may stabilize a mechanism that safeguards a pool of competent cells at the early stages of the boundary development. Together, these observations indicate the existence of at least three phases of AM development: first, the establishment of a cell pool that is competent to form AMs (P1–P2); second, the maintenance of such cells in a cellular environment of differentiation (P3–P4); and third, the activation of the AM initiation pathway (P5–P6). This view is consistent with the detached meristem hypothesis, which proposes that a few pluripotent cells detach from the primary SAM and associate with the developing leaf axils (Steeves and Sussex, 1989).

Ectopic expression of *Sde1A* enhances leaf dissection and the formation of EMs at the distal leaflet boundaries (Figure 4, D–I). In contrast, *sde* mutants show reduced leaf dissection and do not form EMs, indicating that *Sde1A* promotes these morphogenetic activities. These processes are restricted to tissues that share several features with the leaf axil (Wang et al., 2016), suggesting that Sde1A requires a specific cellular environment and/or the availability of specific interaction partners to exert its function. One such candidate, which is expressed in the leaf axil and the leaflet boundary, is the AM regulator Ls that can interact with Sde1A.

In contrast to the role of *Sde1A* in AM initiation, rice *lax2-1*, and maize *ba2-3112* mutants show defects in AM maintenance during the vegetative phase. During reproductive development, *lax2-1* and *ba2-3112* mutants show AM formation defects that were not observed in *sde1a* mutants. These phenotypes correlate with the accumulation of *LAX2* mRNA in vegetative SAMs and developing AMs, and of *LAX2* and *ba2* mRNA in IM and reproductive AMs. The closest *LAX2/ba2* homolog in tomato, *Sde1B* (Supplemental Figure S7A), is expressed in the SAM, similar to *LAX2/ba2*; however, no AM initiation or maintenance defects are present in the *sde1b* mutant, suggesting the existence of redundant functions by homologous genes or differences in the mode of AM regulation in tomato compared to rice and maize. However, it is important to note that the *sde1b* mutant shows a similar pattern of misexpressed genes as in the *sde1a* mutant, and both Sde1A and Sde1B interact with Lhp1 and Ls. Besides the differences in expression patterns between the related genes in rice, maize, and tomato, the differences in phenotypic output may indicate different requirements for Sde1 activity at the SAM and in the boundary region: High levels of Sde1 may be required in the leaf axil prior to the establishment of new meristems, whereas low levels of Sde1 may be needed for SAM maintenance. The closest *Sde1A* homologs in rice and maize are Os12g0479100 and GRMZM2G447297 (Supplemental Figure S7A), which might potentially perform activities similar to *Sde1A* during AM initiation.

### *Sde1* probably originated from a *Bmi1* gene that lost its RING finger-encoding domain in the last common ancestor of embryophytes

An *Sde1*-like gene first appeared in embryophytes, with *Marchantia polymorpha* containing a single-copy gene. The existence of paralogs in several plant species (Figure 5A) suggests that *Sde1* then underwent successive rounds of gene duplications followed by retention, probably due to whole-genome duplications in the ancestors of mosses, spermatophytes, and monocotyledons (Paterson et al., 2004; Rensing et al., 2007; Jiao et al., 2011; Vanneste et al., 2014). A previous phylogenetic examination of proteins with RAWUL domains did not detect Sde1 proteins (Sanchez-Pulido et al., 2008). The phylogenetic reconstruction analysis presented here shows that the Sde1 clade clusters with plant Bmi1 proteins in a group that is distinct from the animal Bmi1 clade and the Ring1 and Wdr48 clades of animals and plants. Sde1 proteins lack the N-terminal RING-finger domain present in Bmi1 proteins. However, the phylogenetic relationships and structural gene conservation in the RAWUL domain-encoding region (Figure 5B;Supplemental Figure S11B) suggest that *Sde1* is derived from a *Bmi1* gene that lost its C3HC4 RING finger-coding region in the last common ancestor of embryophytes, but not in the last common ancestor of seed plants, as postulated by Yao et al. (2019).

Similar to animal and plant BMI1 paralogs, those of Sde1 show low sequence conservation in the RAWUL domain. Because the RAWUL domain is implicated in protein–protein interactions (Kim, 2017), this may imply that variation exists in the interacting partners recruited by Sde1-like proteins, as observed for Bmi1 proteins from humans and mouse (Gao et al., 2012; Fursova et al., 2019; Scelfo et al., 2019).

### Sde1A cooperates with BMI1 proteins

In nonplant model systems, PRC1 activity maintains cellular pluripotency and drives differentiation in a tissue-specific manner by the formation of various PRC1 complexes with different enzymatic activities (Aloia et al., 2013; Loubiere et al., 2019). In tomato, the *Bmi1*-related gene *Sde1A* is required to maintain high expression levels of the meristem regulators *TKn2* and *CLV3* and the boundary genes *Ls*, *Bl*, and *Gob* (Supplemental Figure S13B). Furthermore, 74% of the misexpressed genes in *sde* are upregulated (≥two‐fold, *P *≤* *0.05, Supplemental Data Set S2), suggesting a repressive function for Sde1A. In the sensitized (VF36) tomato background, *Sde1A* functions redundantly with *Bmi1A* to promote leaf dissection and EM formation, whereas in the nonsensitized (Moneymaker) background, they cooperate to promote AM formation. By contrast, *Bmi1C* counteracted *Sde1A* and *Bmi1A* activity in the VF36 background. This result demonstrated that in tomato, various *Bmi1* genes possess opposing roles for the regulation of morphogenetic processes, likely as a result of their differential recruitment to target loci (Figure 6; Supplemental Figure S13). Moreover, Sde1A interacts with the PRC1 component Lhp1 (Supplemental Figure S12), but not with RING1 proteins, suggesting that Sde1A may compete for Lhp1 binding with Bmi1 or that it may form noncanonical PRC1 (ncPRC1) complexes, whose activity is independent of ubiquitylation. In mouse embryonic stem cells, Bmi1 paralogs affect target genes in a manner that can be dependent or independent of RING1 and H2A ubiquitylation (Fursova et al., 2019; Scelfo et al., 2019). In addition, canonical PRC1 (cPRC1) and ncPRC1 complexes cooperatively repress target genes (Fursova et al., 2019; Scelfo et al., 2019). Similarly, the interplay between different *BMI1* genes and *Sde1A* in tomato indicates that cPRC1 and ncPRC1 complexes may also exist in plants. These complexes may promote an epigenetic environment in the center of boundary domains that favors the maintenance of cells with morphogenetic competence, to allow AM formation, leaflet formation, and EM formation (Figure 7G).

### Sde1A and Ls are components of a common genetic pathway

Recruitment of PRC1 complexes to target genes is achieved via several mechanisms, one of which involves transcription factors (Merini and Calonje, 2015). In tomato, the transcription factor gene *Ls* is expressed in a band-shaped domain in leaf axils (Busch et al., 2011), which overlaps with that of *Sde1A* (Figures 3, D and E, 7, A–D). In addition to the pronounced phenotypic similarity between *sde* and *ls* mutants, *sde1a* does not enhance the *ls-1* phenotype. Furthermore, Sde1A and Ls proteins interact in yeast and in planta (Figure 7, C and D), suggesting that Sde1A and Ls participate in a common pathway, in which Ls might guide Sde1A to target genes. The expression patterns and phenotypic features of individual mutants indicate that both genes also possess independent activities. For example, *Ls* promotes organ separation (Greb et al., 2003; Rossmann, 2013), whereas *Sde1A* modulates the reproductive transition of sympodial shoots (Supplemental Figures S3 and S18).

The expression domain of *Bl*, which encodes another regulator of AM initiation, overlaps with that of *Ls* and *Sde1A*; however, *sde1a* and *bl-2* mutants show an additive genetic interaction in a *Sde1A* dose-dependent manner. Double *sde1a bl-2* mutants are strongly defective in AM formation, with 61% of plants producing no side shoots. This observation demonstrates that *Sde1A* and *Bl* take part in independent pathways that modulate AM initiation during vegetative as well as reproductive development. In rice and maize, the AM regulators LAX1 and BA1 interact with the closest Sde1B homologs LAX2 and BA2 (Supplemental Figure S7). In contrast to LAX1/BA1, the tomato *LAX1/ba1* putative ortholog *Uf* neither regulates axillary branching in the vegetative phase nor interacts genetically with *Sde1A* (Supplemental Figure S19). Taken together, these results suggest that in tomato, the Uf pathway is not a major regulator of shoot branching. This hypothesis is in contrast to the functions of LAX2/BA2 in rice and maize, respectively, and indicates that the recruitment of *Sde1* and *Uf* homologous gene modules that regulate shoot branching occurs in diverse ways in different plant species.

In contrast to *sde*, but similar to *lax2-1* and *ba2-3112*, tomato mutants compromised in AM formation during vegetative development also show branching defects during reproductive growth. For instance, *ls-1* and *bl-2* possess fewer vegetative AMs and fewer flowers per inflorescence (Pier, 2015). These pleiotropic effects have excluded the use of the reduced shoot branching trait in elite greenhouse tomato lines, which normally require pruning to concentrate resources in a limited number of fruits along a single axis. The specific defects in *sde1a* in vegetative AM formation might represent a valuable tool for breeding tomato cultivars with reduced side-shoot formation.

## Materials and methods

### Plant material, growth conditions, and phenotyping

Seeds of *S. lycopersicum* cv VF36 (LA0490) and the *sde* mutant (in the VF36 background) were a gift from John Yoder (University of California at Davis). Seeds of *S. lycopersicum* cv Moneymaker (LA2706), cv M82 (LA3475), cv Antimold-B (LA3244), cv Lukullus (LA0534), cv Ailsa Craig (LA2838A), *ls-1* mutant (Antimold-B LA0329), *bl-2* mutant (Lukullus LA0980), and the wild species *S. pennellii* (LA0716) were obtained from the Tomato Genetics Resource Center at the University of California at Davis, USA. The *uf-1* mutant (Ailsa Craig near-isogenic line MLE567) was kindly provided by Yuval Eshed (The Weizmann Institute of Science, Israel).

Seeds were treated with saturated trisodium phosphate (Na_3_PO_4_) for 15 min. After washing with water, seeds were kept in water for 3 d in the dark and then sown on pots containing 50% of coco-peat and 50% perlite. Plants were grown under long-day conditions (16-h light/8-h dark) in a greenhouse under natural light supplemented with artificial light from a combination of high-pressure sodium lamps and high-pressure halogen lamps (∼910 µmol m^−2^ s^−1^), a relative humidity of 25%–55%, and day/night temperatures of 30°C/17°C. Controlled long-day conditions (16-h light/8-h dark) were provided in a Bronson walk-in chamber (Bronson Incubator Services B.V.) with Sylvania T12 IRS fluorescent lamps (F65W/33-640 IRS, ∼230 µmol m^−2^ s^−1^), a relative humidity of 45%–55% and day/night temperatures of 25°C/20°C. Plants were drip-irrigated in the greenhouse and manually irrigated in the Bronson walk-in chamber, and were fertilized under standard regimes with an electrical conductivity of 2.2 dS m^−2^, pH= 5.6, NH_4_ to a total N ratio of 0.05, N/K ratio of 1.8, and P/K+Ca ratio of 0.055.

The number of side shoots was recorded for all leaf axils of the primary shoot. The number of leaves on the primary shoot was used as a proxy for flowering time. Inflorescence branching was calculated as the number of flowers on the primary inflorescence. Leaf complexity was represented by the mean number of first- and second-order leaflets from leaf 1 to leaf 8. Internode length represented the mean length of internodes from leaves one to five. Statistical calculations were carried out by two-tailed, two-sample *t* test.

For stereomicroscopy and SEM, plants were grown for 2 weeks (vegetative phase) or 4 weeks (reproductive phase) in a Bronson chamber. Scoring of side shoots and internode length measurements were performed for 8-week-old plants grown in a Bronson chamber or in the greenhouse. For leaf complexity phenotyping, 4-week-old plants were transplanted to 5-L pots with standard soil and were grown in the greenhouse for 8 additional weeks. Ectopic shoots on leaves were counted in plants grown in the greenhouse for 12 weeks.

### Interspecific mapping population

An interspecific F_2_ population derived from a cross between *S. lycopersicum sde* and *S. pennellii* was generated. Plants grown under long-day conditions were phenotyped for the presence of side shoots. One out of 80 plants displayed the *sde* phenotype. Selected F_2_ progeny were backcrossed to *sde* and a BCF2 population was evaluated with markers described in Tanksley et al. (1992).

### DNA sequencing

Total genomic DNA was extracted from leaf tissue using the DNeasy Plant Mini Kit (Qiagen) according to the manufacturer’s instructions. Libraries were prepared according to the Illumina TruSeq DNA (PCR-free) protocol and sequenced on an Illumina HiSeq 2500 platform (Illumina) at the Genome Centre of the Max Planck Institute for Plant Breeding Research, Cologne, Germany. In total, 289,329,010 and 281,617,026 paired-end 100-bp reads were obtained for wt and *sde* samples, respectively. Reads were aligned to the *S. lycopersicum* reference sequence SL4.0 using the QIAGEN CLC genomics server 11.0 (parameters: No masking, match score = 1, mismatch cost = 2, cost of insertions and deletions = Linear gap cost, insertion cost = 3, deletion cost = 3, length fraction = 0.5, similarity fraction = 0.8, global alignment = No, autodetect paired distances = Yes, nonspecific match handling = Map randomly). Out of all reads obtained, 95% were pair-mapped to the reference genome. Variants were called using the fixed ploidy variant detection module (parameters: ploidy = 2, required variant probability (%) = 98.0, ignore positions with coverage above = 100, Ignore broken pairs = Yes, ignore nonspecific matches = Regions, minimum coverage = 20, minimum count = 2, minimum frequency (%) = 98.0, base quality filter = Yes, neighborhood radius = 5, minimum central quality = 20, minimum neighborhood quality = 15, significance (%) = 1.0, remove pyro-error variants = Yes, in homopolymer regions with minimum length = 3, with frequency below = 0.8). Unique *sde* variants were identified by removing *sde* variants that were shared with VF36 (Supplemental Table S1). Variants that changed the amino acid sequence were extracted as candidates that had functional consequences on protein activity (Supplemental Table S4).

### RNA-seq

Shoot apices of vegetative plants including four leaf primordia (10-d-old) were harvested using forceps and a stereomicroscope (Leica MZ-16FA). Forty apices per genotype and four biological replicates were sampled. Total RNA was extracted using the RNeasy Micro Kit (Qiagen) according to the manufacturer’s instructions, followed by removal of DNA with DNase I (Roche). Libraries were prepared according to the Illumina TruSeq stranded RNA protocol and sequenced on an Illumina HiSeq 2500 platform (Illumina) at the Genome Centre of the Max Planck Institute for Plant Breeding Research, Cologne, Germany. In total, 73,206,185 and 76,254,799 single-end 100-bp reads were obtained for wt and *sde* samples, respectively. Reads were aligned to the *S. lycopersicum* reference sequence SL4.0 using the QIAGEN CLC genomics server 11.0 (parameters: mismatch cost = 2, insertion cost = 3, deletion cost = 3, length fraction = 0.8, similarity fraction = 0.8, global alignment = No, strand-specific = both, and maximum number of hits for a read = 10). Out of all reads obtained, 99% aligned to the reference genome. Changes in gene expression were calculated using the differential expression for RNAreq 2.1 tool of the QIAGEN CLC genomics server 11.0 and using the test of Baggerly et al. (2003). *P*-values were corrected using false discovery rate (Supplemental Table S2). Differentially expressed genes between wt and *sde* were identified. Variants were called using the fixed ploidy variant detection module of QIAGEN CLC genomics server 11.0 (parameters: ploidy = 2, required variant probability (%) = 98.0, ignore positions with coverage above = 100,000, ignore nonspecific matches = Reads, minimum coverage = 10, minimum count = 20, minimum frequency (%) = 80.0, base quality filter = Yes, neighborhood radius = 5, minimum central quality = 20, minimum neighborhood quality = 15, significance (%) = 1.0, remove pyro-error variants = Yes, in homopolymer regions with minimum length = 3, with frequency below = 0.8). Unique *sde* variants were extracted by removing *sde* variants that were shared with VF36 (Supplemental Tables S1, S3). Variants that altered the amino acid sequence were extracted as candidates that had functional consequences on protein activity (Supplemental Table S4).

### Tissue collection, RNA extraction, and RT-qPCR

For RT-qPCR analyses, SAMs including leaf primordia one to four were collected. Twenty apices per genotype and three biological replicates were sampled. Total RNA was extracted using the RNeasy Micro Kit (Qiagen) and digested with DNase I (Roche). One microgram of total RNA was used for cDNA synthesis using the SuperScript III First-Strand Synthesis System (Invitrogen). RT-qPCR was carried out using SYBR Green PCR Master Mix (Applied Biosystems) and a CFX 384 Touch Real-Time PCR Detection System (Bio-Rad). The tomato *GAPDH* (Solyc05g014470) gene was used as an internal control (Expósito-Rodríguez et al., 2008). All primer sequences can be found in Supplemental Table S6.

### Complementation

Plasmid constructs were designed as described by Lampropoulos et al. (2013). A genomic DNA fragment including the presumptive *Sde1*A promoter was PCR-amplified with CloneAmp HiFi (TakaRa) from the Bacterial Artificial Chromosome (BAC) clone Sly-B-Hba 129H16 (INRA, France) with *Sde1A* promoter primers and cloned into pGGA000 (Addgene # 48856) using NEBuilder HiFi DNA Assembly (New England Biolabs). The *Sde1*A coding region was amplified in two fragments using VF36 genomic DNA as a template to synonymously mutate a *Bsa*I site. The first fragment was amplified with *Sde1A* genomic 1 primers and the second fragment with *Sde1A* genomic 2 primers. Both PCR fragments were seamlessly assembled into pGGC000. The DNA sequence downstream of the stop codon was amplified with *Sde1A* terminator primers and cloned into pGGE000. The binary vector pAGM4723 was modified to make it compatible with the Greengate cloning system. To this end, a fragment containing the *ccdB+* gene was amplified from pGGZ003 using ccdB+ cassette primers and seamlessly assembled into the *Bbs*I-cut pAGM4723 vector. The modified vector was named pAGM4723GG and deposited at Addgene (#136960). The final complementing construct was assembled using GoldenGate cloning (*Bsa*I) by combining the promoter, genomic and terminator modules with the pGGB003, pGGD002, pGGF008, and pAGM4723GG modules. The *Sde1A-Venus* translational reporter construct was assembled by combining the *Sde1A* promoter, genomic, and terminator modules with the pGGB003, pGGD_RT_li_Venus (Addgene # 136973), pGGF008, and pAGM4723GG modules. The *Lateral suppressor* promoter was amplified from Moneymaker genomic DNA using *Ls* promoter primers and cloned into pGGA000 using NEBuilder HiFi DNA Assembly (New England Biolabs). The *Ls* coding region was amplified using genomic *Ls* primers with Moneymaker genomic DNA as a template, and the PCR fragment was seamlessly inserted into pGGC000. The DNA sequence downstream of the stop codon was amplified in two fragments using Moneymaker genomic DNA as template to synonymously mutate a *Bsa*I site. The first fragment was amplified with *Ls* terminator 1 primers and the second fragment with *Ls* terminator 2 primers. Both PCR fragments were seamlessly assembled into pGGE000. The final *Venus-Ls* construct was assembled as described above by combining the *Ls* promoter, genomic and terminator modules with the pGGB_RT_Venus_li (Addgene # 136973), pGGD002, pGGF008, and pAGM4723GG modules. The corresponding construct was introduced into Agrobacterium (*Agrobacterium tumefaciens*) strain GV3101 and used for Agrobacterium-mediated transformation of the *sde* mutant using young leaf explants (Knapp et al., 1994). The *Ls-Venus* construct was introduced into the *ls-1* mutant by Agrobacterium-mediated transformation. Transgenic plants were tested for presence of the transgene using primers T-DNA Sde1A. All primer sequences can be found in Supplemental Table S6.

### Confocal microscopy

The shoot apices were imaged by confocal microscopy at one time point as described by Burian et al. (2016). Briefly, microscopic observations were carried out using an upright confocal laser-scanning microscope (Leica TCS SP8) with long-working distance water immersion objectives 20×. The cell wall was stained with 0.1% propidium iodide (PI; Sigma-Aldrich) for 3–10 min. Excitation was at 488 nm at 20% full power (and 30% laser output). The image collection was at 505–545 nm for Venus and 600–656 nm for PI.

### Yeast two-hybrid and bimolecular fluorescence complementation

To fuse the Gal4 DNA binding domain or activation domain to the test proteins, Sde1A, sde1a, Sde1A N-terminal deletion, Sde1A C-terminal deletion, Sde1B, Bmi1A, Bmi1B, Bmi1C, Ring1A, Ring1B, Lhp1, Ls, and Bl were amplified from cDNA and seamlessly cloned into pGBKT7 (Clontech) digested with NcoI and BamHI or into pGADT7 (Clontech) digested with EcoRI and BamHI. All primer sequences are listed in Supplemental Data Set S6. Yeast strain AH109 was used for cotransformation with pGBKT7 and pGADT7 constructs encoding the candidate interacting proteins. Yeast colonies were selected on medium lacking leucine and tryptophan. Positive clones were tested further by growing three independent yeast colonies in medium lacking Leu, Trp, and His or lacking Leu, Trp, His, and Ade.

DNA fragments for *Sde1A*, *Lhp1*, *Ls*, *Bl*, and *TKn2* were generated by amplification from cDNA and seamlessly cloned into pDEST-VYCE(R)GW digested with XbaI or into pDEST-VYNE(R)GW digested with XbaI. PCR primers used for cloning are listed in Supplemental Table S6. The resulting plasmids were introduced into Agrobacterium strain GV3101 by electroporation and the bacteria used for Agrobacterium-mediated coinfiltration. Briefly, an overnight culture was used to inoculate a fresh culture that was allowed to grow to an OD600 ∼ 0.8. Then, 2 mL of cells was pelleted at 1,000*g* for 10 min. The cell pellets were resuspended in infiltration medium (5 g L^−1^d-glucose, 50 mM MES (Sigma), 2 mM Na_3_PO_4_, 0.1 mM acetosyringone). This step was repeated twice; cells were then resuspended in 1 mL of infiltration medium. Before coinfiltration, 5–6 weeks old *N. benthamiana* plants were exposed to white light for one h to allow for stomata opening. For co-infiltration, equal volumes of Agrobacterium were mixed and used to infiltrate *N. benthamiana* leaves. Plants were then left overnight in the dark and returned to long day conditions; 5-mm^2^ segments of the infiltrated zone were mounted onto a glass slide covered with water and a coverslip. The interactions were analyzed at 24–48 h post infiltration under a Zeiss LSM 700 confocal microscope.

### CRISPR-Cas9 mutagenesis

Plasmid constructs were designed as described by Belhaj et al. (2013) using the Golden Gate cloning system (Engler et al., 2009). Briefly, two CRISPR RNAs (crRNAs) directed towards the coding sequence of the target gene were designed using the CRISPR-P 2.0 tool (Liu et al., 2017). Single guide RNAs (sgRNAs) were developed by PCR by combining the crRNA and the trans-activating crRNA (tracrRNA) using the pICH86966 plasmid as template. Each pair of sgRNA was placed downstream of the Arabidopsis U6 promoter in the Level 1 acceptors pICH47751 (sgRNA1) and pICH47761 (sgRNA2) plasmids. Primers containing the crRNA target sequences, which were used to clone sgRNA1 and sgRNA2, are listed in Supplemental Table S6. The Level 1 constructs pICH47731-NOSpro:NPTII, pICH47742-35S:Cas9, pICH47751-AtU6pro:SP5G-sgRNA-1, and pICH47761-AtU6:SP5G-sgRNA-2 were assembled into the binary Level 2 plasmid pAGM4723. The corresponding constructs were introduced into Agrobacterium strain GV3101 and used for Agrobacterium-mediated transformation (Knapp et al., 1994). Transgenic plants were tested for the presence of the transgene using Cas9 primers. Mutations in the target sequence were analyzed using the primers listed in Supplemental Table S6 and by Sanger DNA sequencing with the same primers. Homozygous plants carrying mutations and lacking the transgene were selected for phenotypic analysis. Mutants were backcrossed to the corresponding wt and analyzed in the BCF2 for co-segregation of the mutant phenotypes. All primer sequences can be found in Supplemental Table S6.

### RNA in situ hybridization

In-situ hybridization was performed as described by Coen et al. (1990) with slight modifications. Antisense probes were synthesized from PCR products that used cDNA as a template. The T7 promoter was included within the reverse primer and the DIG RNA Labeling Mix from Roche was used to label RNA with digoxigenin-UTP by in vitro transcription with T7 RNA polymerase (Roche). Primer sequences are listed in Supplemental Table S6.

Shoot apices were fixed with 4% paraformaldehyde and 0.03% Tween-20. Plant material was dehydrated using a series of increasing ethanol concentrations without NaCl. Fixed material was embedded in Paraplast (Kendall) in an ASP300 tissue processor (Leica). Probes were not hydrolyzed. Embedded tissue was sectioned (8 μm) and hybridized according to Coen et al. (1990). After the color reaction, slides were mounted in 30% glycerol and photographed using differential interference contrast microscopy.

### Phylogenetic reconstruction

BLASTP searches (Altschul et al., 1997) from public genome and transcriptome databases were performed to retrieve homologous proteins. The databases searched were Phytozome 12 (Goodstein et al., 2011), Metazome 3 (https://metazome.jgi.doe.gov), Gramene (Gupta et al., 2016), Mycocosm (Grigoriev et al., 2013), and NCBI (Johnson et al., 2008). Initially, the RAWUL domains of the proteins Sde1A, Bmi1A, Ring1A, and Wdr48 were used as a query to identify homologs. After the identification of homologs, their RAWUL domains were used to search for related paralogs. To address the relationship between the different RAWUL domains, multiple sequence alignment (Edgar, 2004) followed by a maximum likelihood phylogenetic reconstruction (Felsenstein, 1973) was performed with pairwise distance UPGMA (Michener and Sokal, 1957), hierarchical clustering and a JTT substitution model (Jones et al., 1992). To infer the reliability of the tree, a bootstrap analysis (Felsenstein, 1985) with 1,000 replicas was performed.

## Accession numbers

Sequence data from this article can be found in the GenBank data libraries under the BioProject accession number PRJNA643909. The associated sequence for the *sde1a* mutant allele can be found under accession number MT767035.

## Supplemental data

**Supplemental Figure S1.** *sde* shows variable expressivity in AM formation.

**Supplemental Figure S2.** *sde* shows defects in AM initiation.

**Supplemental Figure S3.** Sympodial shoots in *sde* undergo faster developmental transition.

**Supplemental Figure S4.** Genetic analysis of *sde1a* in three tomato backgrounds.

**Supplemental Figure S5.** *sde* variant distribution per chromosome.

**Supplemental Figure S6.** *Sde1A* coding region and protein sequences.

**Supplemental Figure S7.** Similarity and identity matrix for Sde1-like proteins from tomato, rice, and maize.

**Supplemental Figure S8.** *Sde1A* alleles generated by CRISPR-Cas9 in the VF36 and Moneymaker tomato backgrounds.

**Supplemental Figure S9.** *Sde1A* and *Sde1B* in situ hybridization.

**Supplemental Figure S10.** Ectopic expression of *Sde1A* in VF36.

**Supplemental Figure S11.** RAWUL phylogenetic reconstruction.

**Supplemental Figure S12.** Sde1A interacts with Lhp1.

**Supplemental Figure S13.** Quantitative expression analysis of *Sde1, Bmi1*, and shoot meristem regulation genes.

**Supplemental Figure S14.** Genetic interaction between *sde1a* and *sde1b*.

**Supplemental Figure S15.** *Bmi1* alleles generated by CRISPR-Cas9 technology.

**Supplemental Figure S16.** Genetic interaction between *sde1a, bmi1a*, and *bmi1c.*

**Supplemental Figure S17.** *Ls* interact with *Sde1A, Sde1B*, and *Bmi1B* proteins.

**Supplemental Figure S18.** *sde1a* and *bl-2* mutually enhance each other’s branching defects.

**Supplemental Figure S19.** Genetic interaction between *sde1a* and *uf-1*.

**Supplemental Table S1.** Mutant and transgenic line list.

**Supplemental Data Set S1.** SNPs in *sde*.

**Supplemental Data Set S2.** Differentially expressed genes in *sde*.

**Supplemental Data Set S3.** SNPs in expressed genes in *sde*.

**Supplemental Data Set S4.** SNPs that change amino acid sequence.

**Supplemental Data Set S5.** *Sde1* and *Bmi1* copy number in different species.

**Supplemental Data Set S6.** List of primers used.

**Supplemental Data Set S7.** Summary of statistical analyses.

**Supplemental File S1.** Protein alignment of RAWUL proteins shown in Supplemental Figure S11A.

**Supplemental File S2.** Newick format of the phylogenetic tree shown in Supplemental Figure S11A.

## Supplementary Material

koab121_Supplementary_Data

## Notes

H.L. and K.T. conceived and designed the research; H.L., G.S., and R.T. performed research; H.L., G.S., and K.T. analyzed the data; H.L. and K.T. wrote the manuscript.

The author responsible for distribution of materials integral to the findings presented in this article in accordance with the policy described in the Instructions for Authors (https://academic.oup.com/plcell) is: Hernán López (lopez@mpipz.mpg.de).
