# Supplementary material for: Super determinant1A, a RAWULdomain-containing protein, modulates axillary meristem formation and compound leaf development in tomato
Source: Plant Cell. 2021 May 1;33(7):2412–30. doi: 10.1093/plcell/koab121 (PMC8364250; doi:10.1093/plcell/koab121)
Supplement: koab121_Supplementary_Data [file koab121_supplementary_data.zip › tpc.00574.2020-s01.pdf]

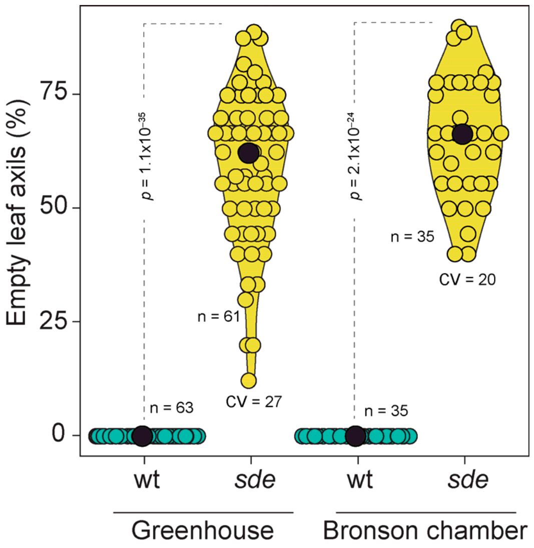

Supplemental Figure S1. *sde* shows variable expressivity in axillary meristem formation.
Violin plots of the shoot branching phenotype (% of empty leaf axils) in wild-type (VF36) and *sde* plants grown in a greenhouse and a Bronson climate chamber. Plants were grown for eight weeks and scored for the presence of axillary buds in the leaf axils of the primary shoot. Median values are indicated by a black circle. n values represent the number of individual plants. *P*-values were determined by two-tailed, two-sample *t*-test for each growth condition. cv represents the coefficient of variation.
(Supports Figure 1)

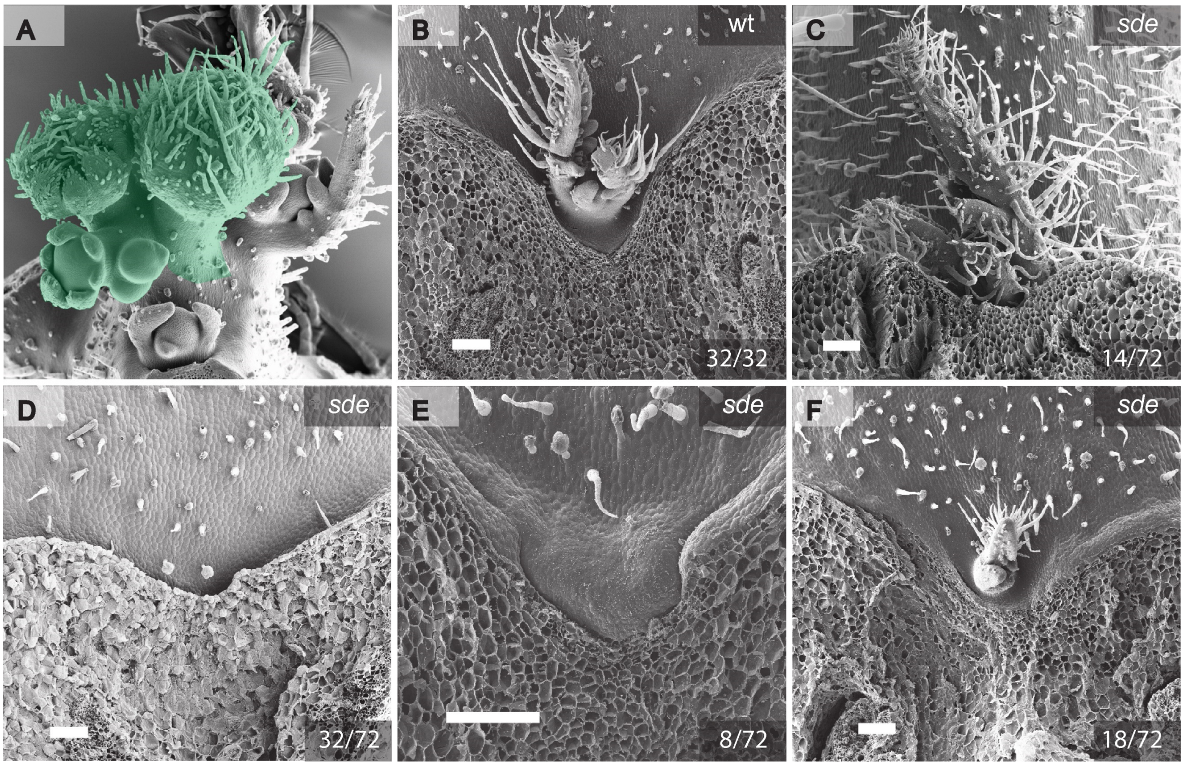

Supplemental Figure S2. *sde* shows defects in axillary meristem initiation.

(**A**) Scanning electron micrographs for leaf axils of 28-day-old plants. A representative primary shoot apical meristem corresponding to an inflorescence (digitally colored in green) is shown, which indicates reproductive growth at the time of scoring for the presence or absence of AMs in wild-type (wt) and *sde* plants.

(**B-F**) Representative leaf axils of wt (**B**) and *sde* (**C-F**) plants indicating AMs (**B, C**), an empty leaf axil (**D**), a disorganized mass of cells (**E**) and an AM delayed in development in comparison to an AM in wt (**F**). The number of observations is indicated in each picture.

(Supports Figure 1)

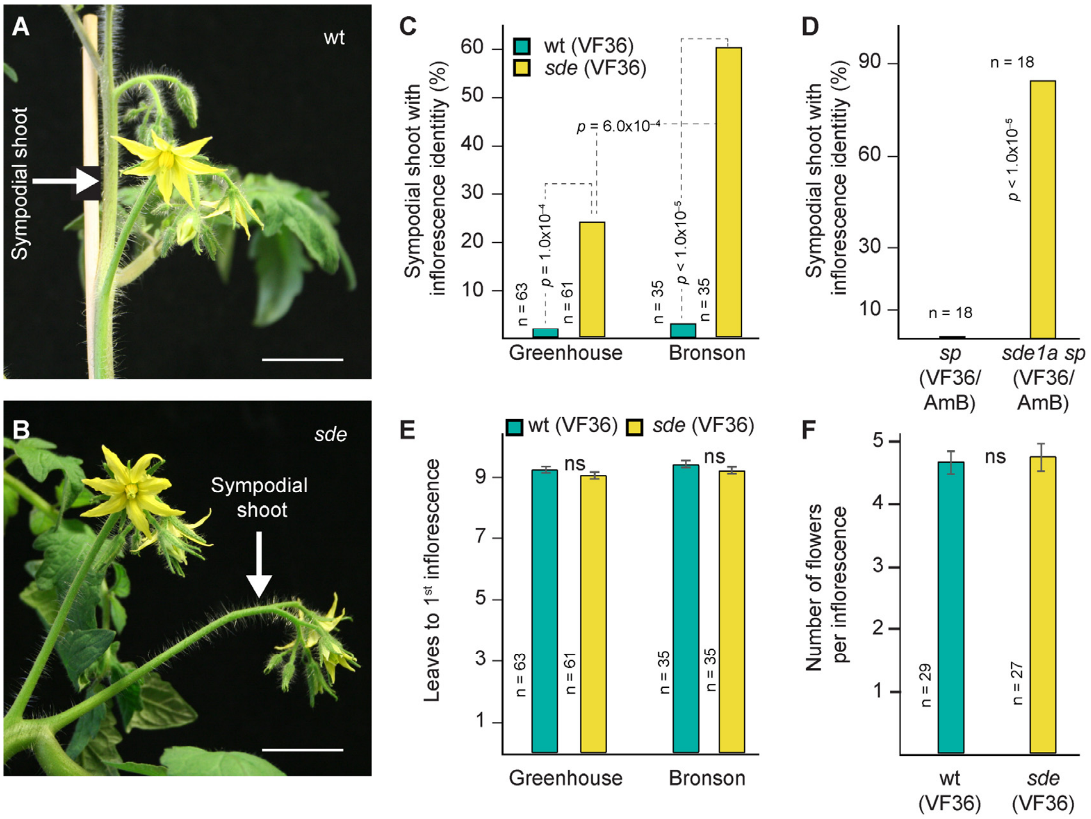

Supplemental Figure S3. Sympodial shoots in *sde* undergo faster developmental transition.

(**A, B**) Developmental stage of the first sympodial shoot in wild-type (wt) (**A**) and *sde* (**B**) plants.

(**C**) Proportion of plants that develop the first sympodial shoot with inflorescence identity in a greenhouse or a Bronson chamber.

(**D**) Proportion of plants that develop the first sympodial shoot with inflorescence identity in a mixed background containing the *sp* mutation alone or in combination with the *sde1a* mutation.

(**E**) Flowering time, measured as the number of leaves produced by the primary shoot of wt and *sde* plants grown in the greenhouse or a Bronson chamber.

(**F**) Number of flowers per inflorescence in wt and *sde* plants grown in greenhouse conditions. n values in **C**, **D**, **E** and **F** represents the number of individual plants. *P* values were determined chi square test in **C** and **D**. ns indicates no significance differences in **E** and **F** as determined by two-tailed, two-sample *t*-test.

(Supports Figure 1)

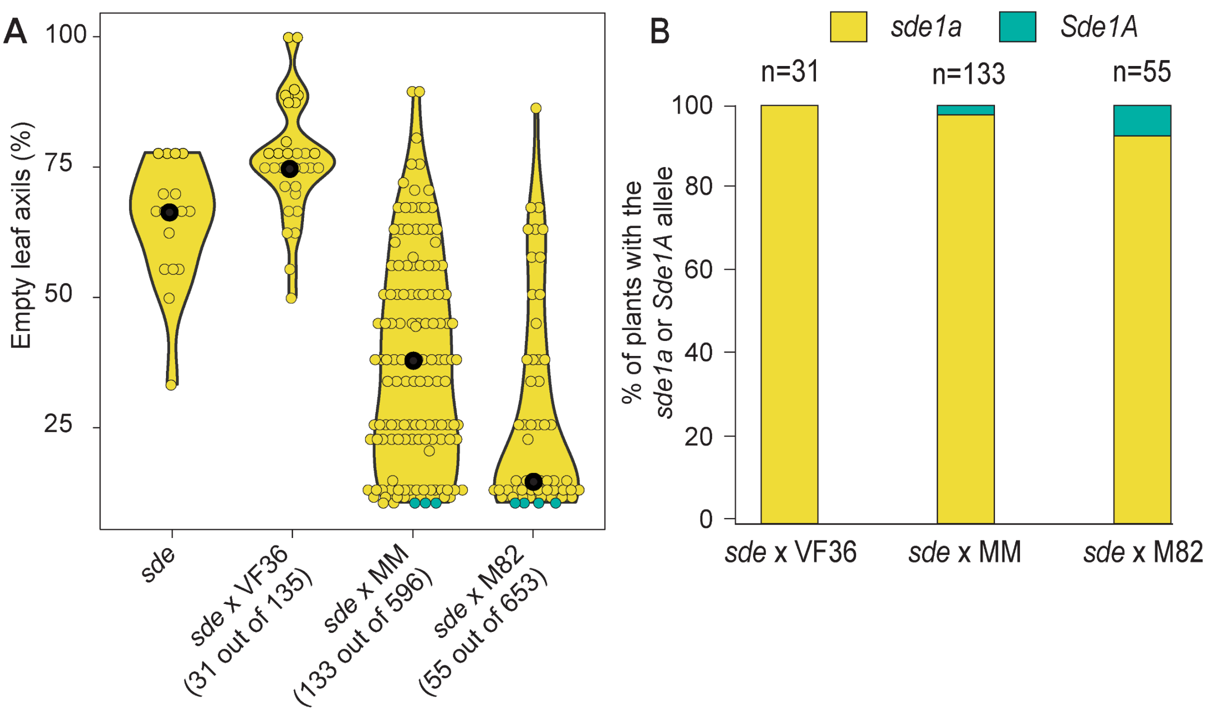

Supplemental Figure S4. Genetic analysis of *sde1a* in three tomato backgrounds.
(**A**) Proportion of empty leaf axils in a *sde* population and 31, 133 and 55 plants that showed at least one empty leaf axils in a backcross of *sde* × VF36 (n = 135), a cross between *sde* x Moneymaker (n = 596) and between *sde* × M82 (n = 653), respectively.
(**B**) Proportion of plants that showed empty leaf axils in **A** and that contained homozygous *sde1a* or *Sde1A* alleles. Heterozygous *Sde1A/sde1a* plants with shoot branching defects were not identified.
(Supports Figure 2)

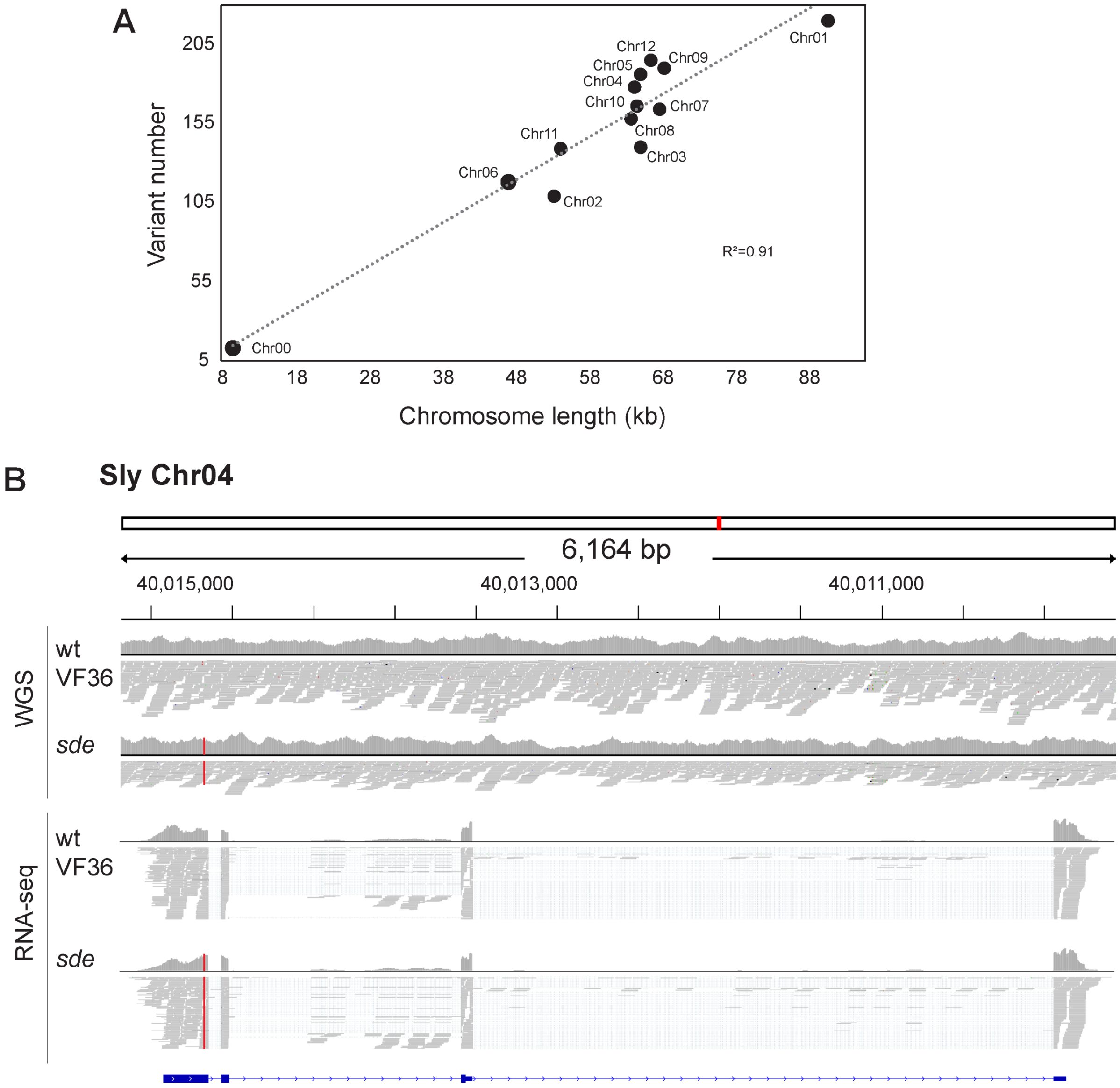

Supplemental Figure S5. *sde* variant distribution per chromosome.

(**A**) Total number of variants in the whole genome sequencing analysis of *sde* compared to VF36. The coefficient of variation (R^2) is indicated.

(**B**) Graphic representation of the WGS and the RNA-seq reads (gray) from wild-type (wt) and *sde* samples mapping to the *Sde1A* locus. The *Sde1A* gene model is shown in blue at the bottom. Red bars indicate a polymorphic site between *sde* and wt.

(Supports Figure 2)

CDS
>*Sde1A:*Solyc04g049190

```
ATGGAGTCAT GTGGATCAGC TATTGAAAGA AGAGGTCACT TTGATGTATT AAAAGAGCAA CAAGGTCAAG AGGAACTAGA GCTAAGATTA
GGGTTAGGAT TAGGGTTAGG TTTAGGATCG GGTGATGATG ATGATATGAA AATGGTGGTT AGAAATAATC ATTTGGGATC TTTGTCTTCG
                                                                            T sde1a
TCTTCGTCTT CATCTTCACT TGTTGTATGC ACTAATACCT CAATTCCACA TTATTCCCCA CCAGGAATTT GGTTCTCTTT ACGTTCCTCA
GTTAATCGGA AAGGAGAATT TCTACCTCAG CTTCCAAAAA CGTTTATAAG AGTCAAAGAC GAGAAGGTGA CAGTCTTCAT GCTTAAAACC
TACCTGGTTA CAAAGCTTGG TCTCTCCAAC CAAGCAGAGG TTGAGATTTC ATGTATGGGG CAAAATTTGA TGCATACTAT AACCTTGAAG
CATGTACGTG ATGCTATTTG GTTGCCAGGA TTAGTAGAAT TCTTGAAGTC AAACACAGAA TTCATTAAAA GTTCACAAGG AGCTAGTCTC
AACTACCTCA TGTCCTTAGACTATGGCAAGA CTTGCCTATG A
```

Protein
>*Sde1A:*Solyc04g049190

```
                                                                                           L sde1a
MESCGSAIER RGHFDVLKEQ QGQEELELRL GLGLGLGLGS GDDDDMKMVV RNNHLGSLSS SSSSSSLVVC TNTSIPHYSP PGIWFSLRSS
VNRKGEFLPQ LPKTFIRVKD EKVTVFMLKT YLVTKLGLSN QAEVEISCMG QNLMHTITLK HVRDAIWLPG LVEFLKSNTE FIKSSQGASL
NYLMSLDYGK TCL*
```

Supplemental Figure S6. *Sde1A* coding region and protein sequences.

Re-annotation of the *Sde1A* coding sequence and the predicted encoded protein sequence. Dark blue and light blue are alternated to indicate the four exons. The *sde1a* mutation is indicated in yellow.

(Supports Figure 2)

A

	Sde1A	Sde1B	LAX2	LAX2-like1	LAX2-like2	BA2	BA2-like1	BA2-like2
Sde1A		23.6	21.8	**26.4**	23.8	19.8	26.9	**28.1**
Sde1B	35.9		**32.6**	31.9	30	**30.8**	30.3	29.8
LAX2 (Os04g0396500)	32.7	**52.5**		36.5	34.3	**46**	35.2	34.7
LAX2-like1 (Os12g0479100)	**40.8**	49	48.2		34.2	33.2	66.8	53.3
LAX2-like2 (Os01g0928400)	40.6	48.7	47.2	50.9		30	32.7	33.9
BA2 (GRMZM2G399641)	29.3	**46.7**	**56.7**	44.4	42.2		32.6	30.9
BA2-like1 (GRMZM2G384695)	44.8	46.4	50	75.2	48.4	44.4		64.1
BA2-like2 (GRMZM2G447297)	**45.1**	45.3	47	65	48.4	40.9	72.8	

B

>LAX PANICLE2 (Os04g0396500)

MVPARSLAHP HPHLVRRRRD HAAAAHGATA RCDDDDDGVV TPRGPTRYMA QEPINHHQHQ HDPPKQPPPR EADDDHHRIQ EREPLPPPTT
TTRNQRLQLQ LGGDGHHNHH HHHHQEVAGT SGSSSGGSSS NNGGGGTRDW LRLATGPASP GASAGSDHDL FPSTTTTAPA PQPPTPTPTP
TPTPTPRHHH HDVLVLPGMP PPGSFLRPGP AMPGIPQASI PTHMLRAAPP WLPPWSPVAA PPPLLPFPHQ HRAFYAAPPT TTPPASSGFD
AIRVVLPPSA VAAAAGVWFV LQAAPLQGRE PFLPQIPRSY LRIKDGRVTV RLLTKYLVNK LGLEDESEVE ITCRGRQLLP ILTLQHVRDS
IWCRRDAVSP SAAPDIPTAD HHQHIMVLQY GRRP

>LAX PANICLE1 (Os12g0479100)

MVTLLRPHRH AHGPAAVDVR RLQLTVFDTR RGRRRSDSSQ HPPPPPPPSP PSSWCFLRSM AQDPSHPHRQ SKDTAAAPPP PPPQEQPQQQ
QPELITAPPP RDDVAHQEPS TSSSSGGGGT DAAGLSLQLG LGPSAPGSPS SSRRKRPRTD AHHEAAAGPS TSAADPQLPE LRLSVLHPGP
GPSSSASAVG AVVAAAPPPP AHEAGTWFVL RAAQNQRREP PLPQIPRSFL RVRDGRMTVR VVMRYLVNKL SLEDDSQLEI TCRGQRLPPE
MTLQHVRDTI WRTPPAEAAA VLPAPGSLST NQVMTLNYGR S

>LAX PANICLE2-like2 (Os01g0928400)

MVPARSRLFH HDSSSSSKRG GGNSSSSCTP QYCIATQLDA AAAAAPARRR HHHHHQLAMA PPEDDAAAGT SSRGGGAGDD DDGDKDWLQL
GLAAAAPTAT APAAAAAPSS STPTAPELDV FAAKQYNGRP PLFPLPILRS YHHHHHHQYG HGGGRCPPPS FFPFARPLRS SGDLPGVMRV
ISPPRRAAAA DAAGLWLTLQ ASPDQFREPI LPQIPKSYLR IKDSNMKVEV VMKYLAGKLG LTQSHLQVEL TCRGQLLPPF LLVKNVRDSI
WCSSLRPSCR EDDDGDGEDD LVELHRRSPA AAATTDHVMA LVYSTSSSCH

>BA2 (GRMZM2G399641)

MCSPPRSSLS NISPPLLAAP CHAATAPSTL PPLATSSAPP LSPARSLSLP SHLISCCSPH PHERSSRPRA VSRLRSPPAR KDRQKGASAQ
ELARRAAAEP GRPGGWNEPA AARMVPARNL QPLARDAHAT PCAAAAGLGV TPTAPMAQEP IDKHHSEPTK PPPPPQERQA DQDTRRHAHA
YGEQQQRTLR PLQPAGVHHQ EAAGSSGSSS NGGAGDWLRL GLSPASPSAG GTQPGLVFAD RAAGPPLLLS SQPQTRTASE ALRPGMGVAP
GPFLHQAAPG IPQASITLPV PRAGPPWLPP WSPSAVAPPL VPFGNRAFYT PGAASGLDAI RVVLPPSAVA GVWFALQAAP HQGREPFLPQ
IPRSYLRIKD ARVTVRLLSK YLAGRLGLED ESEVEITCRG RQLPAFLTLQ HVRDGIWCQG DAAAASPSVV PDMSAANHHH LMVLQYGRRS

>BA2-like1 (GRMZM2G384695)

MVTLLRPHRH AHGPAAVSVR RLSTRHSQHQ LPPPPPLPGP GSVVVVLRPM AQDPSHPHRQ SKDDTAAPPQ QPEIPRDVRE AAASTSASSG
GSDAGSSWLQ LGIGGPSSAS PPPSPPHRLK RPRPDDDAGP SVSVSVQPAA APLLPPPQLQ LQLSLQPGPS SSSSAAPVGA VVAAAPPPPA
HEAGLWFLLR AAQNQRREPP LLQIPRSFLR VREGRMTVRV VMRYLVNKLG LDDDSQLEIT CRGQHLQPTM TLQQVRDTIW RPVPAEAAAV
LPAPGSPSTS QVMTLHYGRC

>BA2-like2 (GRMZM2G447297)

MAQDPSRPHR HSSKDTAAQQ PEPEHQHEHE QQPPAEILHQ LAPQAQPQPP HDVHEPAAAA SSSSSSDAGS SWLQLGIGGP SPSSPPRRKR
PRRDDDDDDA GPSTATSSVQ PAAAPPPQLP PQLLLSLQPA GRPSSSSSSA PAPAPARAVA PPPPAHEAGT WFLLRAAPNQ RREEPPLPHI
PRSYLRVRDG RMMTVRVVMR YLVNKLGLDD DSQLEITCRG QRLQPTMTLQ QVRDTIWRPA VPAEAAAVLP APGSRSTDHI MTLHYCRS

Supplemental Figure S7. Similarity and identity matrix for Sde1-like proteins from tomato, rice and maize.
(**A**) Similarity (green) and identity (blue) matrix values for the full-length tomato Sde1 proteins were determined with MatGAT against the rice and maize homologs.
(**B**) The closest homologs of Sde1A and Sde1B in rice and maize are marked in bold. Rice LAX2 and maize BA2 similarity and identity values are marked in bold. Protein sequence from rice and maize used for comparison to Sde1 proteins. The RAWUL domain is underlined.
(Supports Figure 2)

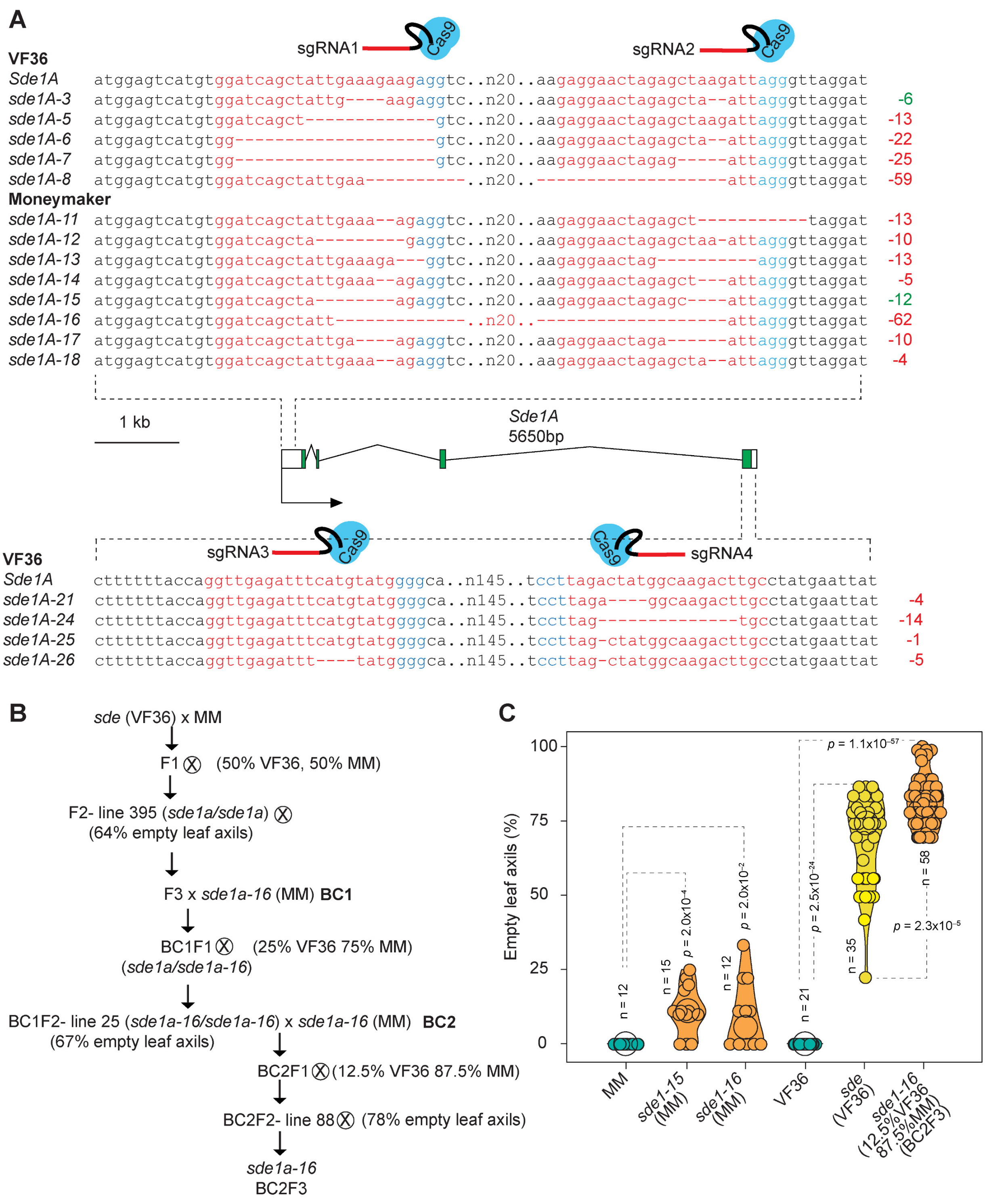

Supplemental Figure S8. *Sde1A* alleles generated by CRISPR-Cas9 in the VF36 and Moneymaker tomato backgrounds.

(**A**) Schematic representation of the *Sde1A* locus indicating the genomic region and the directed mutagenic events targeting the non-conserved (white) or the conserved RAWUL coding (green) regions. CRISPR-Cas9 targeted regions

are indicated above and below the *Sde1A* locus depiction. sgRNA sequences are represented in red followed by the adjacent protospacer motif in blue. Partial DNA sequences of individual deletions in the first and fourth exons are shown. The size of in-frame (green) and frameshift (red) deletions is indicated on the right in bp.
(**B**) Genetic scheme indicating the generation of the *sde1a-16* BC2F3 population.
(**C**) Violin plots of the percentage of empty leaf axils in wild-type (MM and VF36), *sde1a-15*, *sde1a*-16 and *sde1a-16* $BC2F_3$ plants. Median values are indicated by a black circle. n values represent the number of individual plants in **C**. *P*-values were determined by two-tailed, two-sample *t*-tests.
(Supports Figure 2)

A

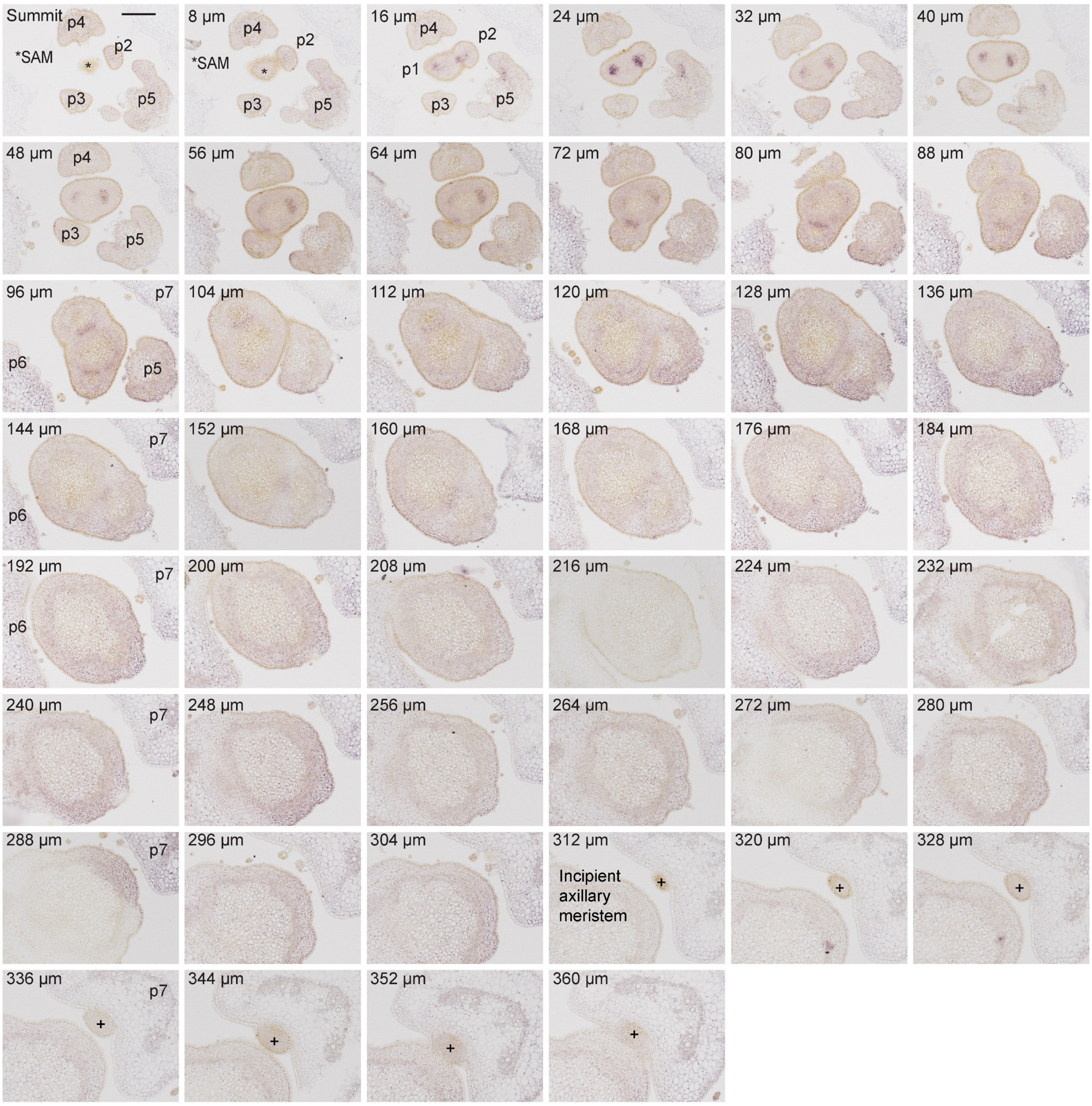

B

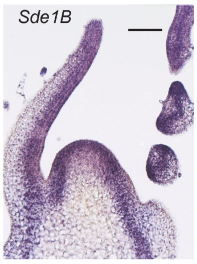

Supplemental Figure S9. *Sde1A* and *Sde1B* in situ hybridization.
(**A**) RNA in situ hybridization analysis of cross-sections through a vegetative shoot apex hybridized with an *Sde1A* antisense probe. Sections cover the summit of the shoot apical meristem (*) to the leaf axil of P7, in which is an AM is developing (+).
(**B**) RNA in situ hybridization analysis of a longitudinal section of a vegetative shoot apex hybridized with an *Sde1B* antisense probe. Bars In **A** and **B** represent 100 µm.
(Supports Figure 3)

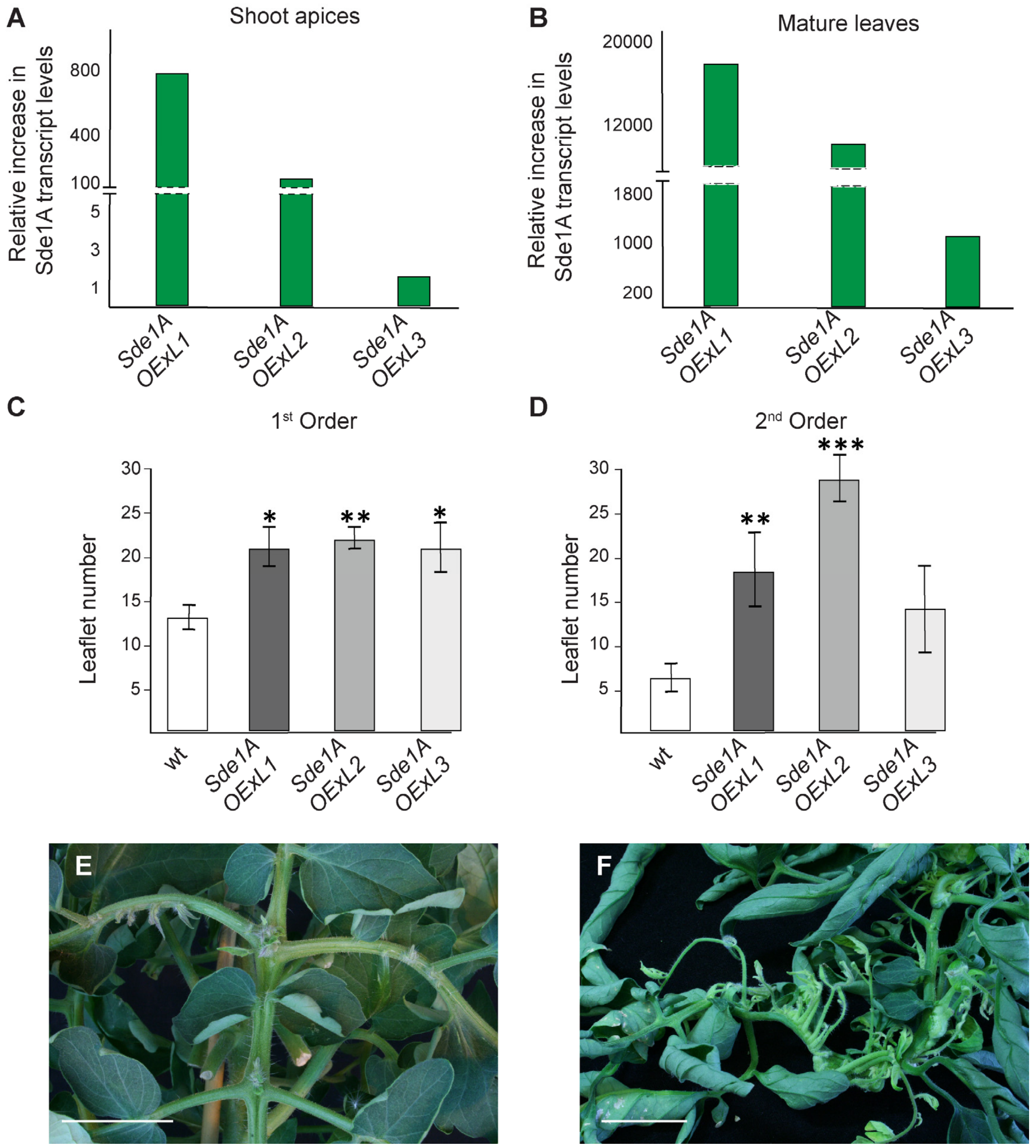

Supplemental Figure S10. Ectopic expression of *Sde1A* in VF36.

(**A, B**) RT-qPCR analysis of relative *Sde1A* levels in shoot apices (two-week-old seedlings, **A**) and leaves of mature plants (eight-week-old, **B**) ectopically expressing *Sde1A* (*Sde1A-OExL*). Expression of *GAPDH* was used as a reference and was set to 1. The increase in *Sde1A* transcript levels in *Sde1A-OExL* lines was compared against wild-type (wt) values.

(**C, D**) Quantification of first-order (**C**) and second-order (**D**) leaflets in wt plants and three selected lines ectopically expressing *Sde1A*.

(**E, F**) Photographs of leaves from lines ectopically expressing *Sde1A* developing ectopic meristems along the rachis and the petiole of the leaves. Bars in **C** and **D** represent the standard error of the mean from five technical replicates. Bars in E and F represent 7 cm. Asterisks represent significance at * *p*-value < 0.05, ** *p*-value < 0.01 and *** *p*-value < 0.001, based on a two tailed two sample *t*-test.

(Supports Figure 4)

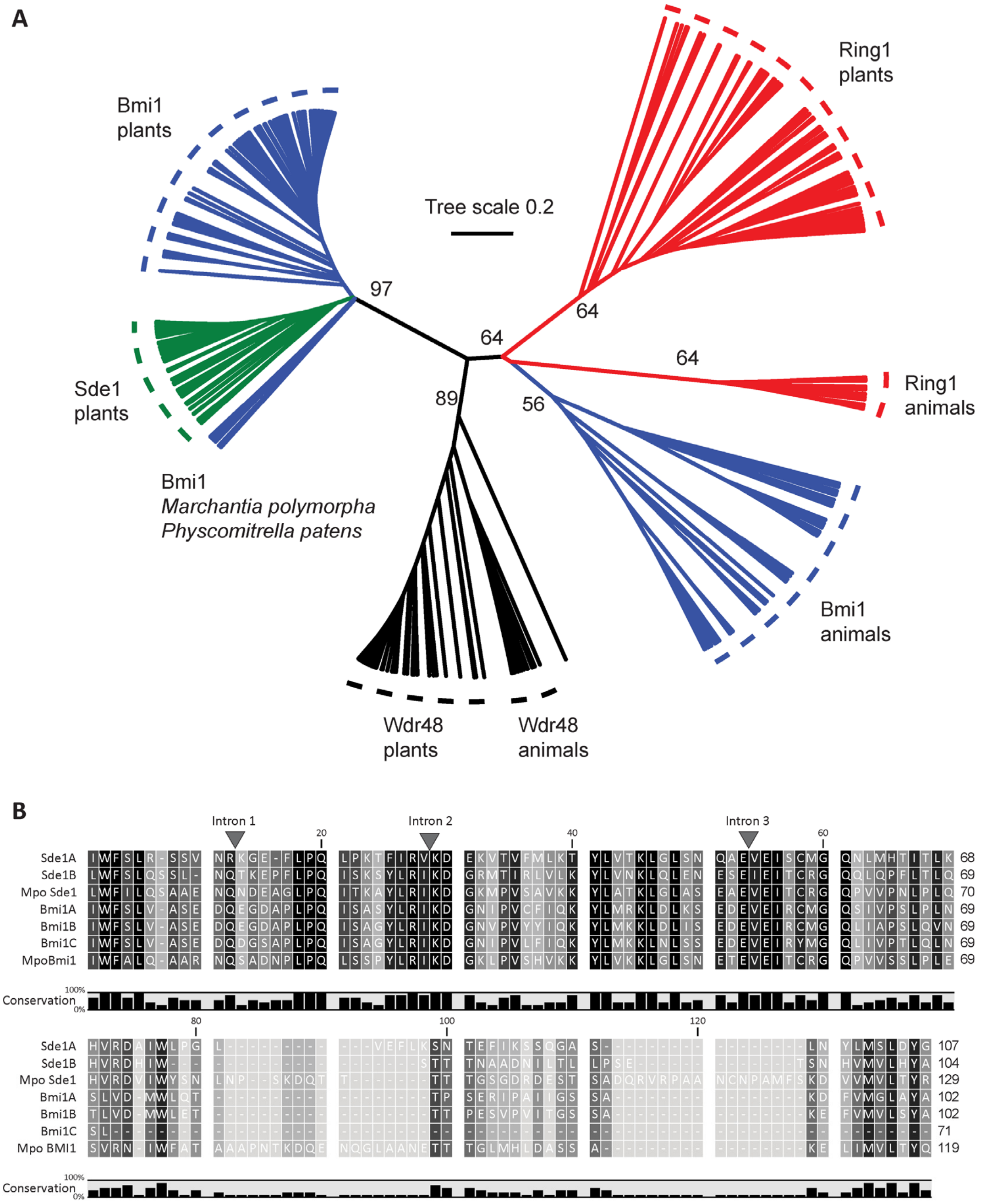

Supplemental Figure S11. RAWUL phylogenetic reconstruction.

(**A**) Phylogenetic clustering of RAWUL domain-containing proteins from animals and plants. The RAWUL domain of Sde1 (green), Bmi1 (blue), Ring1 (red) and Wdr48 (black) proteins was used to infer the relationship between the different protein families. The tree shown is based on a maximum likelihood phylogenetic reconstruction. Bootstrap analysis was performed with 1,000 replicates. Values higher than 50 are indicated at the base of each branch. Bmi1 from basal plants include *Marchantia polymorpha* and *Physcomitrium* (*Physcomitrella*) *patens*.

(**B**) RAWUL domain alignment of Sde1-like and Bmi1-like proteins from tomato and *Marchantia polymorpha*. Conservation from highest (black) to lowest (pale gray) is indicated for each amino acid and as a bar graph on the bottom of each amino acid. The positions of the introns that separate the coding sequence are indicated as an arrowhead.

(Supports Figure 5)

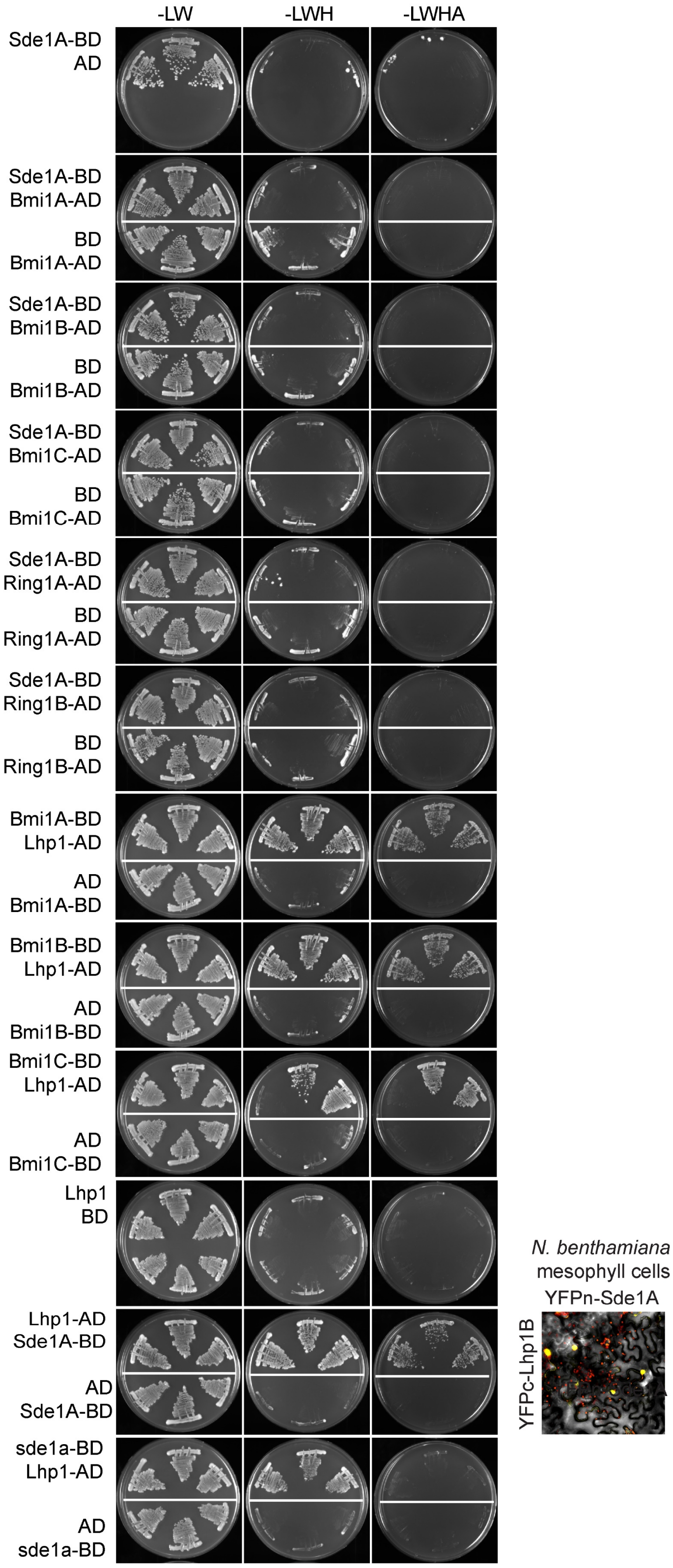

Supplemental Figure S12. Sde1A interacts with Lhp1.

Yeast two-hybrid interaction analysis of Sde1A with Bmi1, Ring1 and Lhp1 proteins. BD denotes the GAL4 DNA-binding domain encoded in the pGBK7 plasmid vector. AD denotes the GAL4 activation domain encoded in the pGADT7 plasmid vector. Selective medium lacking Leucine (–L), Tryptophan (–W), Histidine (–H) or Adenine (–A) are indicated on top. Bimolecular fluorescence complementation (right panel) between Sde1A fused to the N-terminal domain of YFP and Lhp1 fused to the C-terminal domain of YFP proteins showed interaction in five independent biological replicates. For controls, refer to Supplemental Figure 17B. (Supports Figure 5)

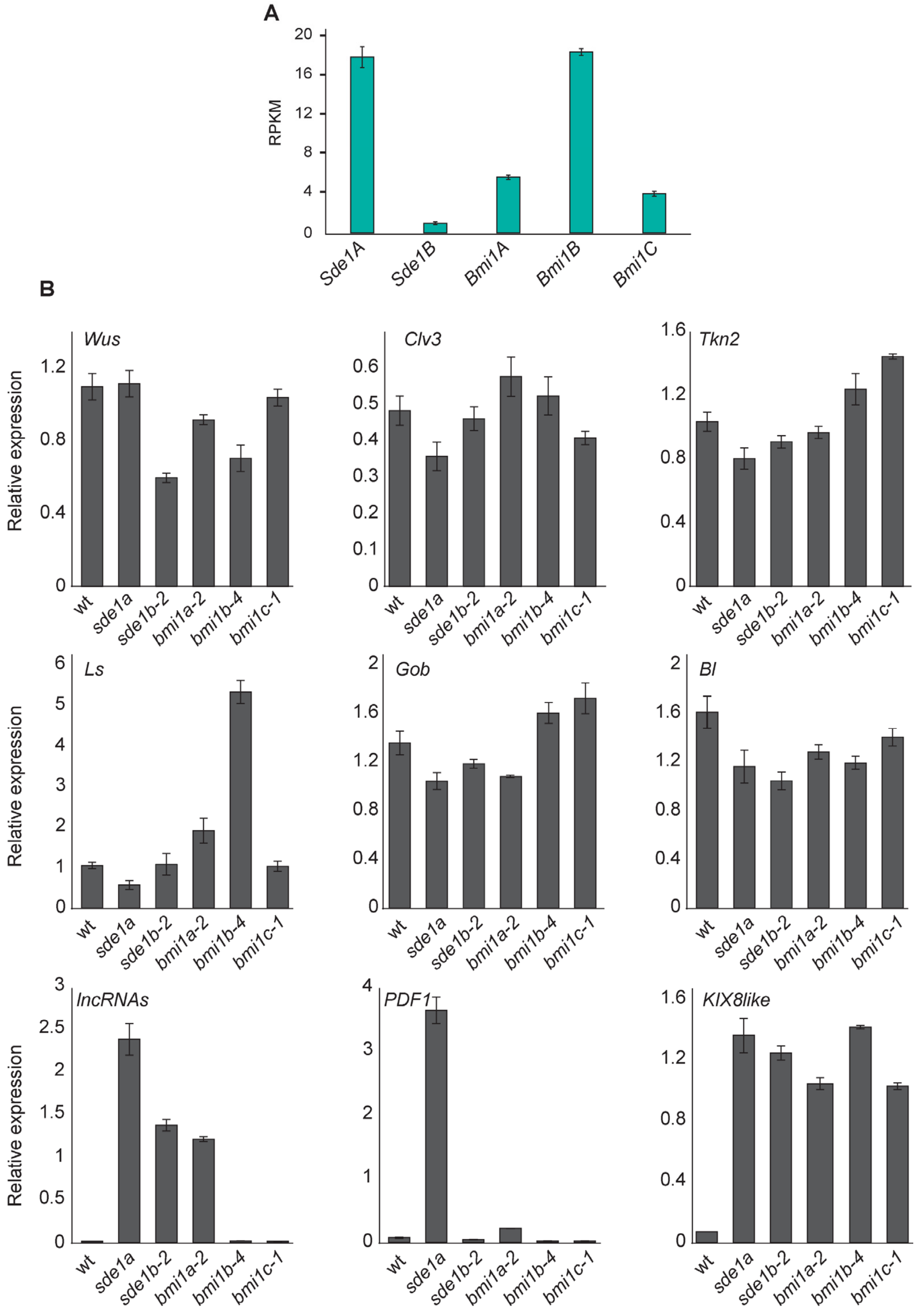

Supplemental Figure S13. Quantitative expression analysis of *Sde1, Bmi1* and shoot meristem regulation genes.

(**A**) Transcript accumulation (RPKM values from RNA-seq experiment) of *Sde1* and *Bmi1* genes in vegetative meristems (two weeks old seedlings).

(**B**) RT-qPCR analysis of *TKn2*, *CLV3*, *WUS*, *Ls*, *Bl*, *Gob, lncRNAs*, *PDF1* and *Kix8like* transcript accumulation in wild-type and mutant vegetative meristems (two weeks old) in (**B**). *GAPDH* transcript accumulation was used as a reference in **B** and was set to 1. Bars in **A** and **B** represent the standard error of the mean from four technical replicates in **A** and three biological replicates in **B**.
(Supports Figure 6)

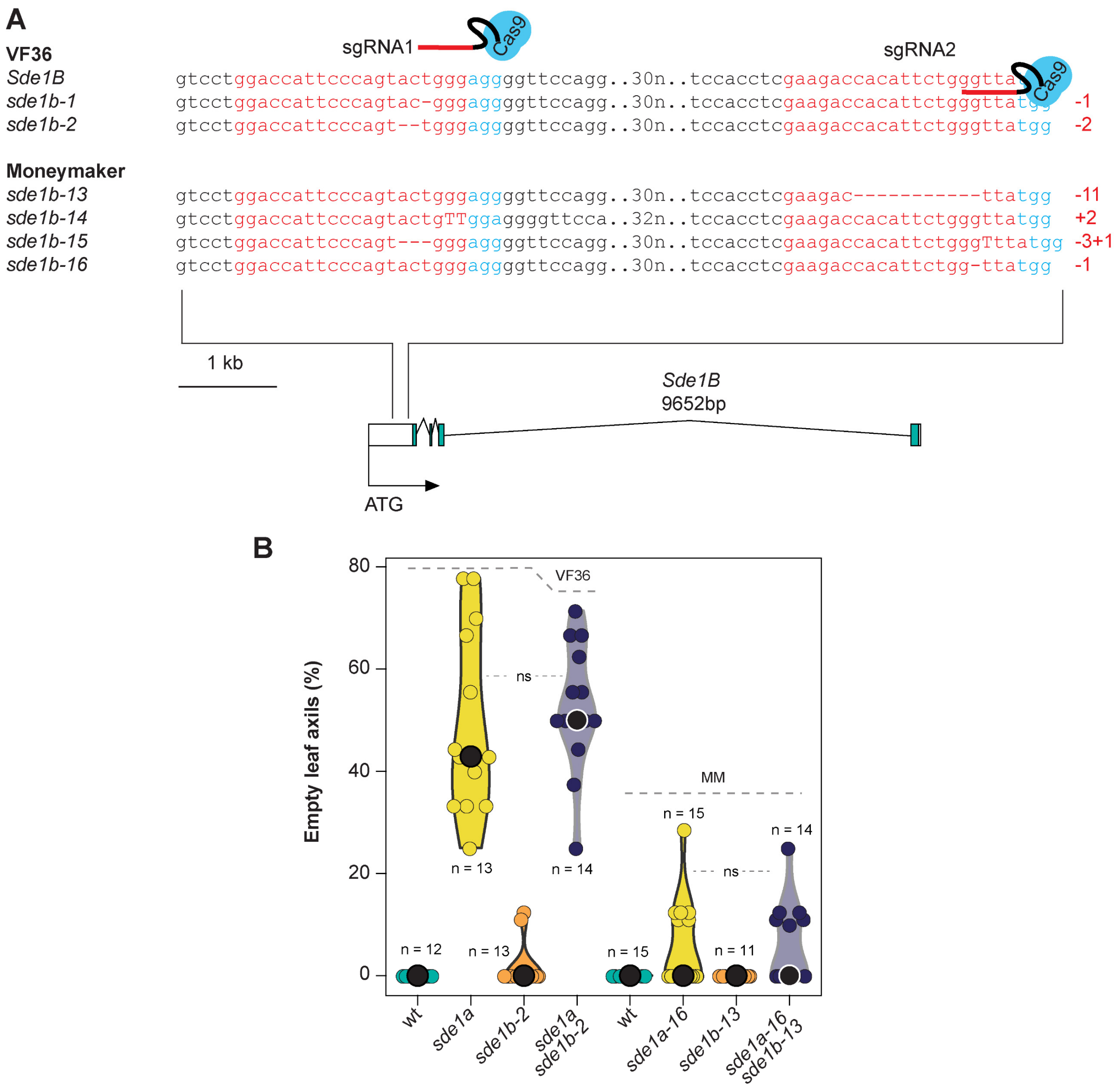

Supplemental Figure S14. Genetic interaction between *sde1a* and *sde1b*.
(**A**) Schematic representation of the *Sde1B* locus. The non-conserved (white) or the conserved RAWUL-coding (green) regions are indicated. The CRISPR-Cas9 targeted regions are indicated above the *Sde1B* schematic. sgRNA sequences are represented in red followed by the adjacent protospacer motif in blue. Partial DNA sequences of individual deletions in the first exon are shown for the VF36 and the Moneymaker tomato backgrounds. The size of frameshift deletions is indicated on the right in bp.
(**B**) Violin plots of the shoot branching phenotypes (% empty leaf axils) of the selected *Sde1B* CRISPR-Cas9-independent mutants in the VF36 and Moneymaker backgrounds. *sde1a sde1b* double-mutant combinations are shown. Median values are indicated by a black or white circle. n values represent the number of individual plants. *P*-values were determined by two-tailed, two-sample *t*-tests.
(Supports Figure 6)

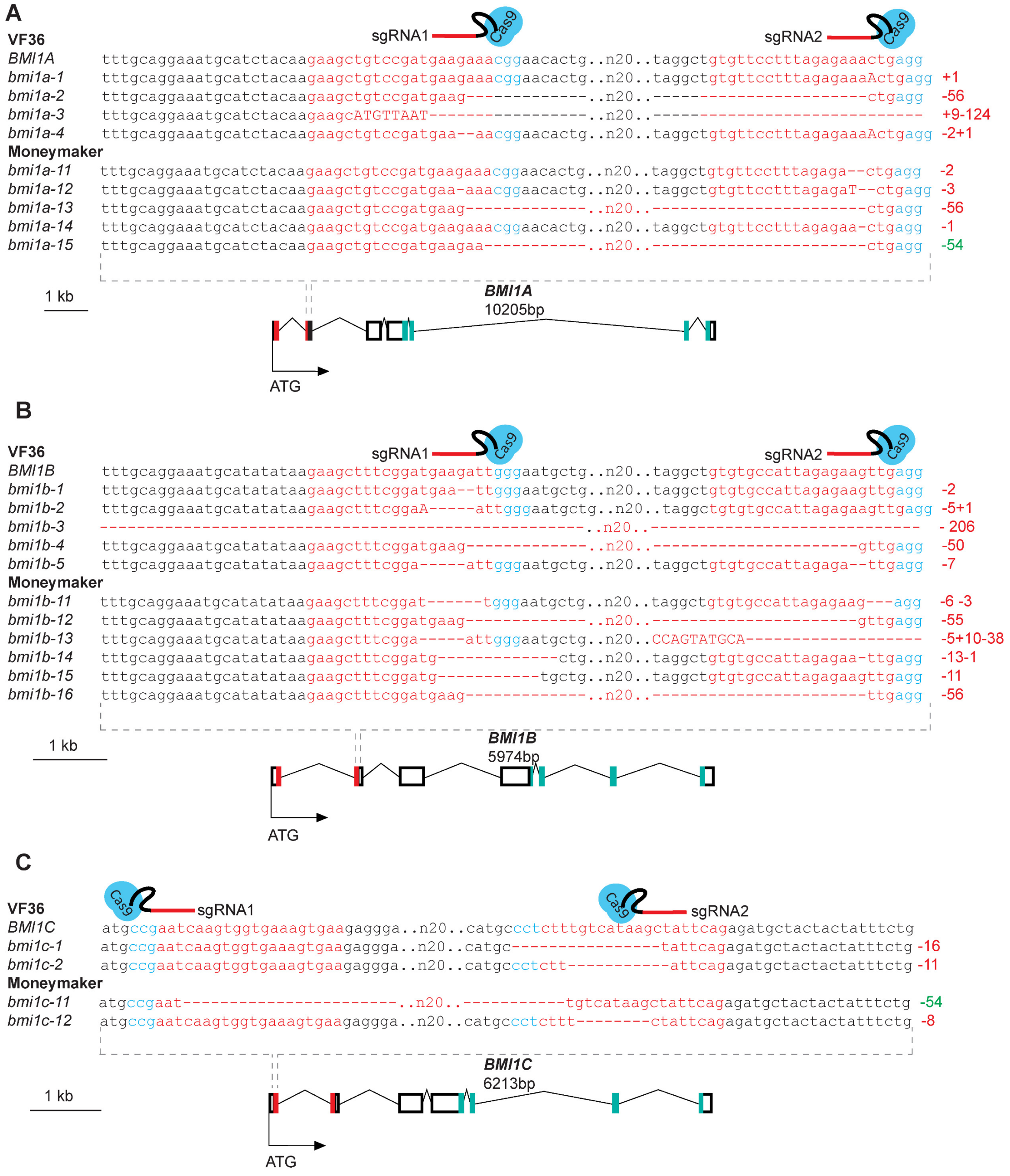

Supplemental Figure S15. *Bmi1* alleles generated by CRISPR-Cas9 technology.

Schematic representation of the *Bmi1A* (**A**), *Bmi1B* (**B**) and *Bmi1C* (**C**) loci. The non-conserved (white) or the conserved RAWUL- (green) and RING- (red) coding regions are indicated. The CRISPR-Cas9 targeted regions are indicated above each *Bmi1* schematic. sgRNA sequences are represented in red followed by the adjacent protospacer motif in blue. Partial DNA sequences of individual deletions are shown for the VF36 and the Moneymaker tomato backgrounds. The size of in-frame (green) and frameshifts (red) deletions is indicated on the right in bp.

(Supports Figure 6)

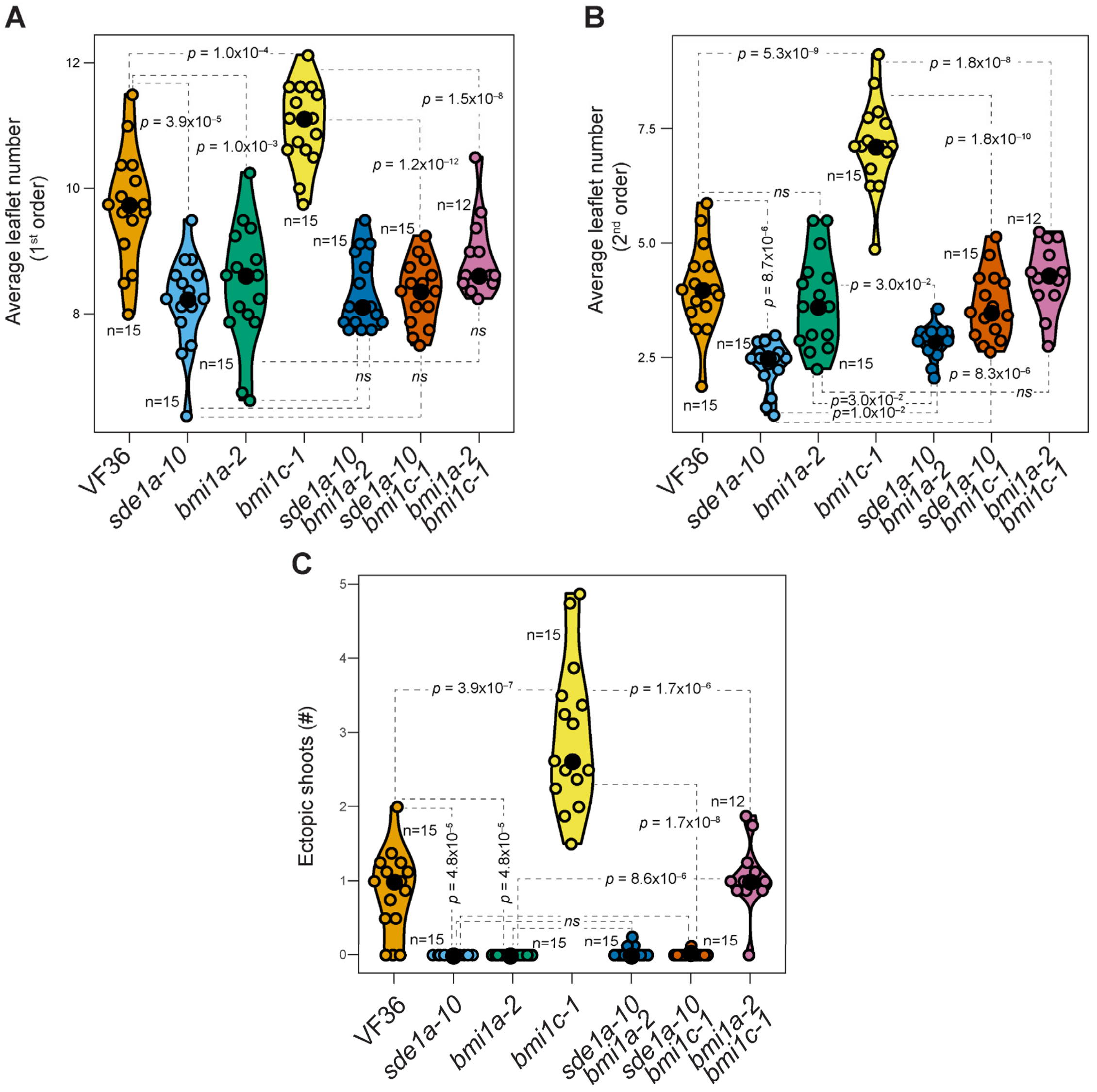

Supplemental Figure S16. Genetic interaction between *sde1a, bmi1a* and *bmi1c*.

(**A, B**) Violin plots of the number of first-order (**A**) and second-order (**B**) leaflets of wt, *sde1a-10*, *bmi1a-2*, *bmi1c-1*, *sde1a-10 bmi1a-2*, *sde1a-10 bmi1c-1* and *bmi1a-2 bmi1c-1* plants.

(**C**) Violin plot of the mean number of ectopic shoots in wt, *sde1a-10*, *bmi1a-2*, *bmi1c-1*, *sde1a-10 bmi1a-2*, *sde1a-10 bmi1c-1* and *bmi1a-2 bmi1c-1* plants. Median values are indicated by a black circle. n values represent the number of individual plants. *P*-values were determined by two-tailed, two-sample t-tests.

(Supports Figure 6)

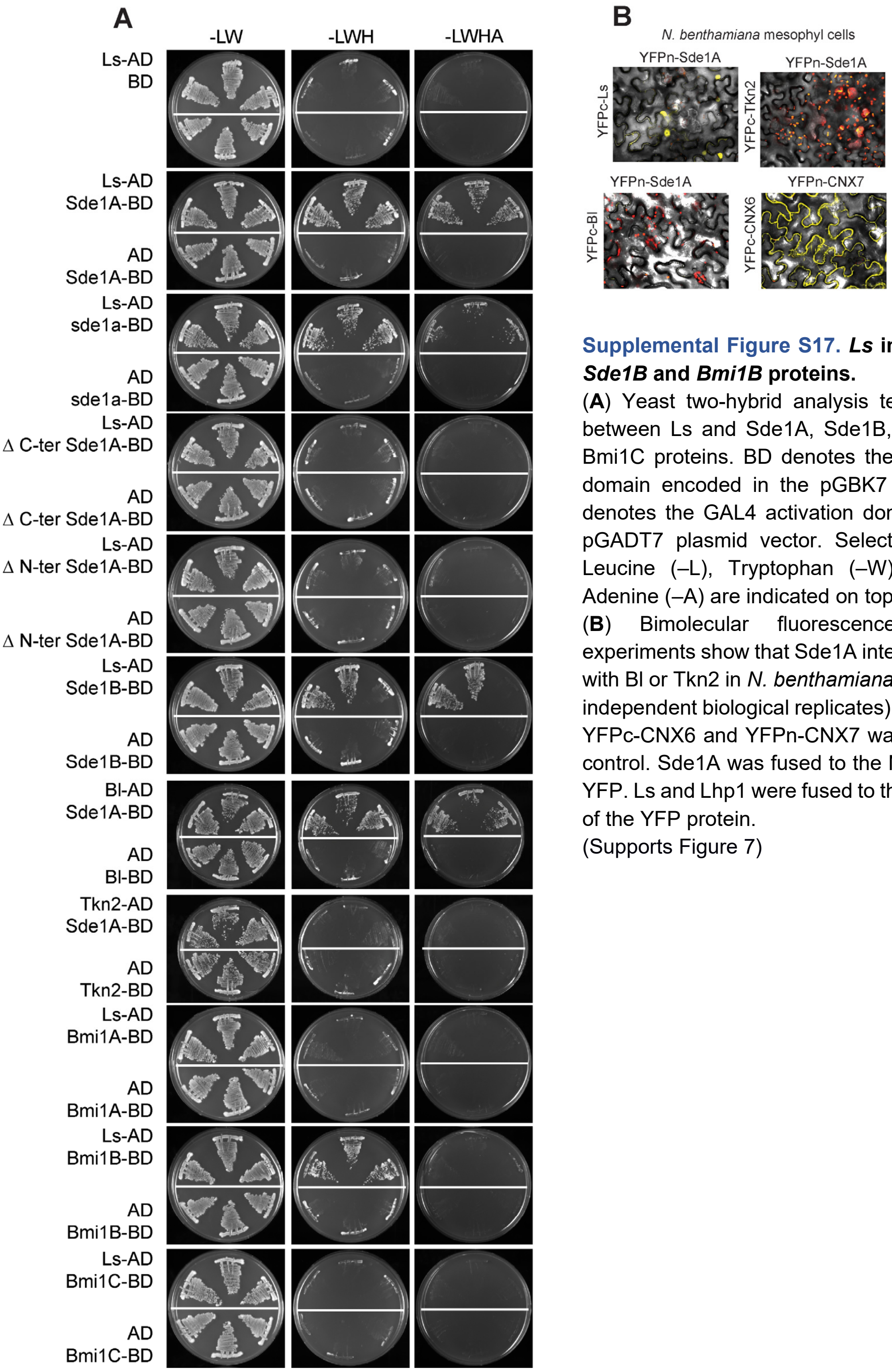

Supplemental Figure S17. *Ls* interact with *Sde1A, Sde1B* and *Bmi1B* proteins.

(**A**) Yeast two-hybrid analysis testing for interaction between Ls and Sde1A, Sde1B, Bmi1A, Bmi1B and Bmi1C proteins. BD denotes the GAL4 DNA-binding domain encoded in the pGBK7 plasmid vector. AD denotes the GAL4 activation domain encoded in the pGADT7 plasmid vector. Selective medium lacking Leucine (–L), Tryptophan (–W), Histidine (–H) or Adenine (–A) are indicated on top.

(**B**) Bimolecular fluorescence complementation experiments show that Sde1A interacts with Ls, but not with Bl or Tkn2 in *N. benthamiana* epidermal cells (five independent biological replicates). Interaction between YFPc-CNX6 and YFPn-CNX7 was used as a positive control. Sde1A was fused to the N-terminal domain of YFP. Ls and Lhp1 were fused to the C-terminal domain of the YFP protein.

(Supports Figure 7)

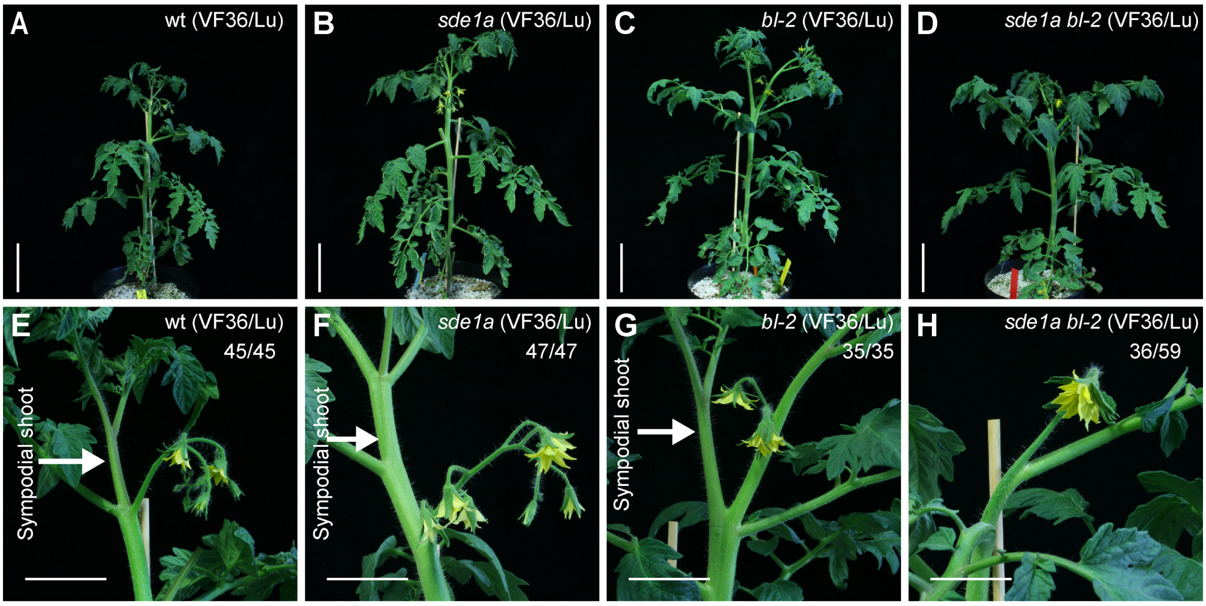

Supplemental Figure S18. *sde1a* and *bl-2* mutually enhance each other's branching defects.
(**A-D**) Growth habit of wild-type (wt, **A**), *sde1a* (**B**), *bl-2* (**C**) and *sde1a bl-2* (**D**) mutants 8 weeks after sowing.
(**E-H**) Close-up of inflorescence and sympodial shoots of wt (**E**), *sde1a* (**F**), *bl-2* (**G**) and *sde1a bl-2* (**H**) mutants. The number of observations is indicated in each figure. Bars in **A** to **D** represent 15 cm, bars in **E** to **H** represent 10 cm. (Supports Figure 7)

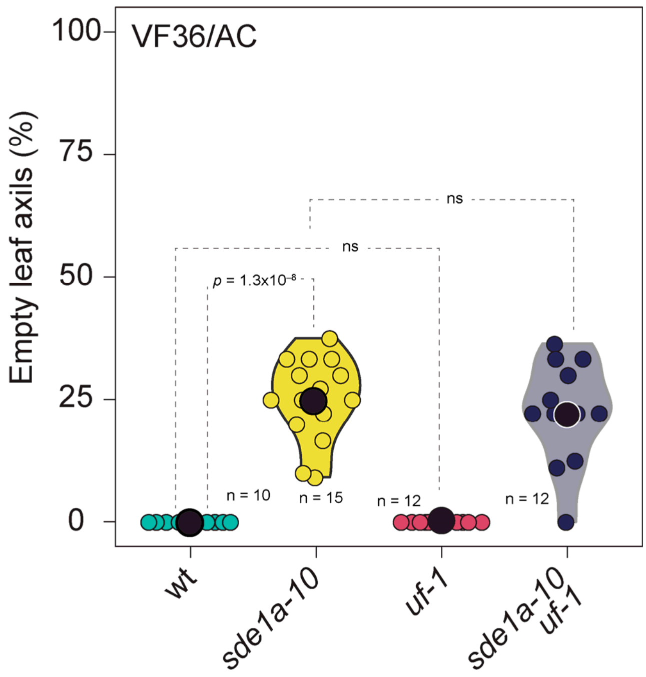

Supplemental Figure S19. Genetic interaction between *sde1a* and *uf-1*.
Comparison of shoot branching phenotypes (violin plots of % empty leaf axils) of the *sde1a-10* and *uf-1* mutants in single and double-mutant combinations. Median values are indicated by a black or white circle. n values represent the number of individual plants. *P*-values were determined by two-tailed, two-sample *t*-test.
(Supports Figure 7)

Supplemental Table S1. Mutant and transgenic line list.

Genotype	Description
sde	Tomato mutant compromised in axillary meristem formation
Sde1A-Venus	Translational reporter line expressing an Sde1A-Venus protein fusion driven by the presumptive *Sde1A* promoter in the tomato VF36 *sde* genetic background. Three independent transgenic events were tested.
Venus-Ls	Translational reporter line expressing a Venus-Ls protein fusion driven by the presumptive *Ls* promoter in the tomato Antimold B *ls-1* genetic background. One transgenic event was tested
sde1a-2	Mutant allele generated by CRISPR-Cas9 mediated genome editing of the gene *Sde1A* in the tomato VF36 genetic background
sde1a-3	Mutant allele generated by CRISPR-Cas9 mediated genome editing of the gene *Sde1A* in the tomato VF36 genetic background
sde1a-5	Mutant allele generated by CRISPR-Cas9 mediated genome editing of the gene *Sde1A* in the tomato VF36 genetic background
sde1a-6	Mutant allele generated by CRISPR-Cas9 mediated genome editing of the gene *Sde1A* in the tomato VF36 genetic background
sde1a-7	Mutant allele generated by CRISPR-Cas9 mediated genome editing of the gene *Sde1A* in the tomato VF36 genetic background
sde1a-8	Mutant allele generated by CRISPR-Cas9 mediated genome editing of the gene *Sde1A* in the tomato VF36 genetic background
sde1a-9	Mutant allele generated by CRISPR-Cas9 mediated genome editing of the gene *Sde1A* in the tomato VF36 genetic background
sde1a-10	Mutant allele generated by CRISPR-Cas9 mediated genome editing of the gene *Sde1A* in the tomato VF36 genetic background
sde1a-11	Mutant allele generated by CRISPR-Cas9 mediated genome editing of the gene *Sde1A* in the tomato Moneymaker genetic background
sde1a-12	Mutant allele generated by CRISPR-Cas9 mediated genome editing of the gene *Sde1A* in the tomato Moneymaker genetic background
sde1a-13	Mutant allele generated by CRISPR-Cas9 mediated genome editing of the gene *Sde1A* in the tomato Moneymaker genetic background
sde1a-14	Mutant allele generated by CRISPR-Cas9 mediated genome editing of the gene *Sde1A* in the tomato Moneymaker genetic background
sde1a-15	Mutant allele generated by CRISPR-Cas9 mediated genome editing of the gene *Sde1A* in the tomato Moneymaker genetic background
sde1a-16	Mutant allele generated by CRISPR-Cas9 mediated genome editing of the gene *Sde1A* in the tomato Moneymaker genetic background
sde1a-17	Mutant allele generated by CRISPR-Cas9 mediated genome editing of the gene *Sde1A* in the tomato Moneymaker genetic background

Genotype	Description
sde1a-18	Mutant allele generated by CRISPR-Cas9 mediated genome editing of the gene *Sde1A* in the tomato Moneymaker genetic background
sde1a-21	Mutant allele generated by CRISPR-Cas9 mediated genome editing of the gene *Sde1A* in the tomato VF36 genetic background
sde1a-23	Mutant allele generated by CRISPR-Cas9 mediated genome editing of the gene *Sde1A* in the tomato VF36 genetic background
sde1a-24	Mutant allele generated by CRISPR-Cas9 mediated genome editing of the gene *Sde1A* in the tomato VF36 genetic background
sde1a-25	Mutant allele generated by CRISPR-Cas9 mediated genome editing of the gene *Sde1A* in the tomato VF36 genetic background
sde1a-26	Mutant allele generated by CRISPR-Cas9 mediated genome editing of the gene *Sde1A* in the tomato VF36 genetic background
sde1b-1	Mutant allele generated by CRISPR-Cas9 mediated genome editing of the gene *Sde1B* in the tomato VF36 genetic background
sde1b-2	Mutant allele generated by CRISPR-Cas9 mediated genome editing of the gene *Sde1B* in the tomato VF36 genetic background
sde1b-13	Mutant allele generated by CRISPR-Cas9 mediated genome editing of the gene *Sde1B* in the tomato Moneymaker genetic background
sde1b-14	Mutant allele generated by CRISPR-Cas9 mediated genome editing of the gene *Sde1B* in the tomato Moneymaker genetic background
sde1b-15	Mutant allele generated by CRISPR-Cas9 mediated genome editing of the gene *Sde1B* in the tomato Moneymaker genetic background
sde1b-16	Mutant allele generated by CRISPR-Cas9 mediated genome editing of the gene *Sde1B* in the tomato Moneymaker genetic background
bmi1a-1	Mutant allele generated by CRISPR-Cas9 mediated genome editing of the gene *Bmi1A* in the tomato VF36 genetic background
bmi1a-2	Mutant allele generated by CRISPR-Cas9 mediated genome editing of the gene *Bmi1A* in the tomato VF36 genetic background
bmi1a-3	Mutant allele generated by CRISPR-Cas9 mediated genome editing of the gene *Bmi1A* in the tomato VF36 genetic background
bmi1a-4	Mutant allele generated by CRISPR-Cas9 mediated genome editing of the gene *Bmi1A* in the tomato VF36 genetic background
bmi1a-11	Mutant allele generated by CRISPR-Cas9 mediated genome editing of the gene *Bmi1A* in the tomato Moneymaker genetic background
bmi1a-12	Mutant allele generated by CRISPR-Cas9 mediated genome editing of the gene *Bmi1A* in the tomato Moneymaker genetic background
bmi1a-13	Mutant allele generated by CRISPR-Cas9 mediated genome editing of the gene *Bmi1A* in the tomato Moneymaker genetic background
bmi1a-14	Mutant allele generated by CRISPR-Cas9 mediated genome editing of the gene *Bmi1A* in the tomato Moneymaker genetic background

Genotype	Description
bmi1a-15	Mutant allele generated by CRISPR-Cas9 mediated genome editing of the gene *Bmi1A* in the tomato Moneymaker genetic background
bmi1b-1	Mutant allele generated by CRISPR-Cas9 mediated genome editing of the gene *Bmi1B* in the tomato VF36 genetic background
bmi1b-2	Mutant allele generated by CRISPR-Cas9 mediated genome editing of the gene *Bmi1B* in the tomato VF36 genetic background
bmi1b-3	Mutant allele generated by CRISPR-Cas9 mediated genome editing of the gene *Bmi1B* in the tomato VF36 genetic background
bmi1b-4	Mutant allele generated by CRISPR-Cas9 mediated genome editing of the gene *Bmi1B* in the tomato VF36 genetic background
bmi1b-5	Mutant allele generated by CRISPR-Cas9 mediated genome editing of the gene *Bmi1B* in the tomato VF36 genetic background
bmi1b-11	Mutant allele generated by CRISPR-Cas9 mediated genome editing of the gene *Bmi1B* in the tomato Moneymaker genetic background
bmi1b-12	Mutant allele generated by CRISPR-Cas9 mediated genome editing of the gene *Bmi1B* in the tomato Moneymaker genetic background
bmi1b-13	Mutant allele generated by CRISPR-Cas9 mediated genome editing of the gene *Bmi1B* in the tomato Moneymaker genetic background
bmi1b-14	Mutant allele generated by CRISPR-Cas9 mediated genome editing of the gene *Bmi1B* in the tomato Moneymaker genetic background
bmi1b-15	Mutant allele generated by CRISPR-Cas9 mediated genome editing of the gene *Bmi1B* in the tomato Moneymaker genetic background
bmi1b-16	Mutant allele generated by CRISPR-Cas9 mediated genome editing of the gene *Bmi1B* in the tomato Moneymaker genetic background
bmi1c-1	Mutant allele generated by CRISPR-Cas9 mediated genome editing of the gene *Bmi1C* in the tomato VF36 genetic background
bmi1c-2	Mutant allele generated by CRISPR-Cas9 mediated genome editing of the gene *Bmi1C* in the tomato VF36 genetic background
bmi1c-11	Mutant allele generated by CRISPR-Cas9 mediated genome editing of the gene *Bmi1C* in the tomato Moneymaker genetic background
bmi1c-12	Mutant allele generated by CRISPR-Cas9 mediated genome editing of the gene *Bmi1C* in the tomato Moneymaker genetic background
sde c	Transgenic line containing an Sde1A genomic fragment driven by the presumptive *Sde1A* promoter in the tomato VF36 *sde* genetic background. 15 independent transgenic events were tested.
